# mTOR may interact with PARP-1 to regulate visible light-induced parthanatos in photoreceptors

**DOI:** 10.1186/s12964-019-0498-0

**Published:** 2020-02-17

**Authors:** Yi-Ran Pan, Jing-Yao Song, Bin Fan, Ying Wang, Lin Che, Si-Ming Zhang, Yu-Xin Chang, Chang He, Guang-Yu Li

**Affiliations:** 1 Department of Ophthalmology, Second Hospital of JiLin University, No.218 Zi-Qiang St, ChangChun, 130041 China; 2 Department of Hemooncolog, Second Hospital of JiLin University, ChangChun, 130041 China; 3 Department of Orthopedics, Second Hospital of JiLin University, ChangChun, 130041 China; 4 Department of Genetics,Basic, Medical College of Jilin University, ChangChun, 130041 China

**Keywords:** PARP-1, mTOR, SIRT1, AIF, Parthanatos, Retinal neuroprotection

## Abstract

**Background:**

Excessive light exposure is a detrimental environmental factor that plays a critical role in the pathogenesis of retinal degeneration. However, the mechanism of light-induced death of retina/photoreceptor cells remains unclear. The mammalian/mechanistic target of rapamycin (mTOR) and Poly (ADP-ribose) polymerase-1 (PARP-1) have become the primary targets for treating many neurodegenerative disorders. The aim of this study was to elucidate the mechanisms underlying light-induced photoreceptor cell death and whether the neuroprotective effects of mTOR and PARP-1 inhibition against death are mediated through apoptosis-inducing factor (AIF).

**Methods:**

Propidium iodide (PI)/Hoechst staining, lentiviral-mediated short hairpin RNA (shRNA), Western blot analysis, cellular fraction separation, plasmid transient transfection, laser confocal microscopy, a mice model, electroretinography (ERG), and hematoxylin-eosin (H & E) staining were employed to explore the mechanisms by which rapamycin/3-Aminobenzamide (3AB) exert neuroprotective effects of mTOR/PARP-1 inhibition in light-injured retinas.

**Results:**

A parthanatos-like death mechanism was evaluated in light-injured 661 W cells that are an immortalized photoreceptor-like cell line that exhibit cellular and biochemical feature characteristics of cone photoreceptor cells. The death process featured over-activation of PARP-1 and AIF nuclear translocation. Either PARP-1 or AIF knockdown played a significantly protective role for light-damaged photoreceptors. More importantly, crosstalk was observed between mTOR and PARP-1 signaling and mTOR could have regulated parthanatos via the intermediate factor sirtuin 1 (SIRT1). The parthanatos-like injury was also verified in vivo, wherein either PARP-1 or mTOR inhibition provided significant neuroprotection against light-induced injury, which is evinced by both structural and functional retinal analysis. Overall, these results elucidate the mTOR-regulated parthanatos death mechanism in light-injured photoreceptors/retinas and may facilitate the development of novel neuroprotective therapies for retinal degeneration diseases.

**Conclusions:**

Our results demonstrate that inhibition of the mTOR/PARP-1 axis exerts protective effects on photoreceptors against visible-light–induced parthanatos. These protective effects are conducted by regulating the downstream factors of AIF, while mTOR possibly interacts with PARP-1 via SIRT1 to regulate parthanatos.

**Video Abstract**

**Graphical Abstract:**

Schematic diagram of mTOR interacting with PARP-1 to regulate visible light-induced parthanatos. Increased ROS caused by light exposure penetrates the nuclear membrane and causes nuclear DNA strand breaks. PARP-1 detects DNA breaks and synthesizes PAR polymers to initiate the DNA repair system that consumes a large amount of cellular NAD+. Over-production of PAR polymers prompts the release of AIF from the mitochondria and translocation to the nucleus, which leads to parthanatos. Activated mTOR may interact with PARP-1 via SIRT1 to regulate visible light-induced parthanatos. 
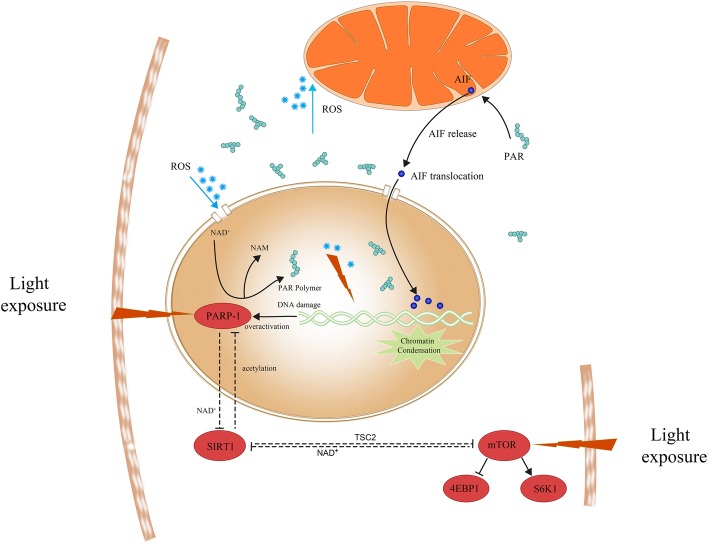

## Background

The death of photoreceptor cells is an important pathological feature of retinal degeneration diseases including age-related macular degeneration (AMD), retinitis pigmentosa (RP), and Stargardt disease that can all ultimately lead to severe vision loss and irreversible blindness [1, 2]. Photoreceptor cells are a specialized type of neuroepithelial cell located in the outer layer of the retina that are capable of visual phototransduction [3]. Photoreceptors are biologically important because they can sense visible electromagnetic radiation light at wavelengths between 400 nm and 700 nm and then transform light signals into nerve impulses that are eventually transmitted from the optic nerve to the brain, thereby forming an image [4]. The initial stage of the visualization process requires photoreceptor proteins in the cell, like rhodopsin and opsin, in order to absorb photons and trigger a change in the cell membrane potential [5].. However, excessive light exposure may cause severe damage to photoreceptors and has previously been used as a model for investigating retinal degeneration [6, 7]. Excessive light exposure is a detrimental environmental factor and plays a critical role in the pathogenesis of retinal degeneration, especially for AMD [8, 9]. Indeed, excessive visible light exposure is a risk factor for exudative AMD [10], and intense or sustained light exposure may damage photoreceptors and exacerbate non-exudative AMD [11]. The pathology of AMD features photoreceptor degeneration similar to that observed following intense light exposure in albino rodents. In addition, a clear correlation between light exposure-induced damage and late non-permeable AMD has been demonstrated in various studies [12, 13]. Therefore, animal retinal light damage models are widely used as models of AMD. In RP, the function and structure of normal photoreceptors are greatly compromised due to various gene mutations, although excessive light exposure might represent a secondary problem that accelerates the death of photoreceptors and prompts disease progression [14]. Thus, it is essential to understand the molecular mechanisms underlying retinal light injury when developing therapeutic strategies to mitigate retinal degeneration diseases.

Previous studies have shown that intensive light exposure can produce photothermal, photomechanical, and photochemical damage of the retina [4]. Among these, photochemical damage could play a predominant role in retinal light injury. During this process, light photons are absorbed by the chromophores of photoreceptors, resulting in energy or electron transfer to other molecules that may then cause photooxidative stress [15]. Additionally, retinal pigment epithelial (RPE) cells engulf the photoreceptor outer segment (POS) and phagocytose the shedding POS membranes to maintain the physiological function of photoreceptor cells, which then results in the continuous excessive accumulation of high levels of reactive oxygen species (ROS) [16]. These processes result in physiological reactions, especially after exposure to intensive light, that may ultimately lead to the overload of oxidative-stress in mammalian retinas once the internal anti-oxidative system is compromised. Moreover, excessive ROS may penetrate the nuclear membrane and cause nuclear DNA strand breaks, thereby triggering cell death cascades.

Poly (ADP-ribose) polymerase-1 (PARP-1) is a 116 kDa protein with three main domains: 1) a 42 kDa N-terminal domain with two zinc finger motifs and a nuclear localization sequence for DNA-binding, 2) a central 16 kDa auto-modification domain, 3) a 55 kDa C-terminal catalytic domain containing the NAD-binding site and the poly (ADP-ribose) (PAR)-synthesizing domain [17]. PARP-1 constitutes a DNA base excision-repair system that senses DNA strand nicks and promotes their repair by synthesizing the PAR polymer. In addition, PARP-1 is involved in the regulation of cell homeostasis and maintenance of genomic stability under physiological conditions [18]. However, excessive PAR produced by the over-activation of PARP-1 in the cytoplasm increases mitochondrial outer membrane permeability and induces the release of inner mitochondrial proteins. The synthesis of large amounts of PAR consumes a large amount of intracellular 5′-adenylate triphosphate (ATP) and nicotinamide adenine dinucleotide (NAD^+^), resulting in the energetic collapse of cells [19]. A previous study demonstrated that PARP-1 was over-activated in the retina of retinal degeneration 1 (rd1) mice, a genetic-modified animal model that mimics hereditary photoreceptor degeneration, while massive accumulation of PAR was also detected in the nuclei of photoreceptors undergoing programmed death [20]. Other gene-modified animal models for evaluating retinal degradation include S334ter and P23H RHO (histidine at position 23 of the rhodopsin gene) rats. In these models, the over-activation of PARP-1 was observed in the retina and was accompanied by increased oxidative stress-induced DNA damage and the accumulation of the PAR polymer [21].

Dawson et al. defined PARP-dependent death as a unique form of cell death known as parthanatos [22]. The typical symptoms of parthanatos include PARP-1 overactivation, PAR accumulation, nuclear translocation of the mitochondrial protein apoptosis-inducing factor (AIF), and large-scale DNA cleavage. In parthanatos, free PAR polymer or poly (ADP-ribosyl) ated acceptor proteins are produced by the overactivation of PARP-1 and are then translocated to the cytosol and mitochondria where they may lead to externalization of phosphatidylserine and dissipation of the mitochondrial membrane potential. AIF is a mitochondrial intermembrane flavoprotein that is synthesized in the cytoplasm as a 67 kDa precursor containing a predicted mitochondrial localization signal at its N-terminal presequence. During its translocation into the mitochondria, AIF is cleaved at the Met53/Ala54 sites into the mature 62 kDa form that then localizes at the inner membrane with its N-terminal exposed to the matrix and its C-terminal exposed to the intermembrane space [23]. Upon stimulation by death, the mature AIF is further processed to a 57 kDa form [24]. The 101-amino-acid residue presequence segment in the intermembrane space is cleaved off by calpains, cathepsins, or both. Overproduced PAR polymer can bind to AIF or increase mitochondrial outer membrane permeabilization (MOMP), thereby favoring the mitochondrial release of the 57 kDa pro-apoptotic AIF form [25, 26]. The 57 kDa AIF guided by a nuclear localization sequence (NLS) further translocates into the nucleus where it causes large-scale fragmentation of DNA, chromatin condensation, and programmed cell death in a caspase-independent manner.

The mammalian/mechanistic target of rapamycin (mTOR) is a serine-threonine protein kinase found in almost all eukaryotic cells that integrates a variety of signals including growth factor levels, oxygen levels, and nutrient and energy availability, in order to regulate protein synthesis and cell growth [27, 28]. mTOR comprises two complexes: the mammalian target of rapamycin complex 1 (mTORC1) and the mammalian target of rapamycin complex 2 (mTORC2). mTORC1 can respond to various stimuli through the PI3K/Akt/mTOR pathway, although Ras and AMPK are involved in the regulation of the mTORC1 signal [29, 30]. The two major downstream factors of mTOR are eukaryotic initiation factor 4E-binding protein 1 (4EBP1) and the p70 ribosomal S6 kinases (S6K1) that are phosphorylated by activated mTORC1. mTOR can regulate protein synthesis through phosphorylation and inactivation of the repressor of mRNA translation, eukaryotic initiation factor 4EBP1, and the phosphorylation and activation of S6K1 [31]. Accumulating evidence has shown that activation of the mTOR signal is widely involved in various retinal degeneration diseases including diabetic retinopathy and age-related macular diseases [32, 33]. mTOR inhibition may also suppress oxidative stress [34], improve dysfunctional mitochondria, and protect photoreceptor cells from degenerative death [35]. Although PARP-1 and mTOR are closely involved in the pathogenesis of retinal degeneration, it is not clear if they are functionally linked, especially in the degenerative death process of photoreceptor cells.

The 661 W cell line is derived from mouse photoreceptors via immortalization with SV40-T antigen expression that is controlled by the promoter of the gene coding for the human retinal receptor-binding protein. This cell line has been a useful model for studying photoreceptor biology in vitro since it exhibits the cellular and biochemical characteristics of cone photoreceptor cells. Such characteristics include the expression of photoreceptor-specific proteins, including blue and green cone pigments, transducin, and cone arrestin [36], but not the proteins specific to retinal pigment epithelial cells. The cell line also serves as a faithful model for photoreceptor cell response to visible light in vitro because they undergo light-induced death as observed in vivo [37] while also exhibiting photoreceptor functions, including retinoid processing. Consequently, the cell line has been widely used to investigate retinal degeneration and neuroprotection in addition to enabling the demonstration of promising therapeutic strategies for these diseases [38].

Previously, we demonstrated that the death of photoreceptors induced by visible light injury mainly occurs via a PARP-1 dependent, but not caspase-dependent, pathway that is involved in AIF activation [39]. In the present study, we further demonstrate that continuous visible light exposure leads to parthanatos-like death in photoreceptor cells. More importantly, we also show that the mTOR signal may interact with PARP-1 to further regulate parthanatos. In addition, our results indicate that sirtuin-1 (SIRT1) (i.e., NAD^+^-dependent deacetylase), which regulates intracellular NAD^+^ levels, may play an important role as an intermediate factor by linking the PARP-1 and mTOR signals. Specifically, SIRT1 inhibition may uncouple the signaling loop between PARP-1 and mTOR. Moreover, light-induced parthanatos-like injury of the retina was further verified with in vivo experiments, while neuroprotection by PARP-1/mTOR inhibition was demonstrated by functional and structural analysis of the retina. Overall, the results of this study provide new insight into the mechanisms of photoreceptor cell death and provide a framework for the development of neuroprotective strategies for retinal degeneration diseases.

## Materials and methods

### Chemicals and reagents

Cell culture media and additives were obtained from the HyClone Company (Beijing, China). Antibodies for mTOR (Cat. No. 32028), p-mTOR (Cat. No. 109268), p-S6K1 (Cat. No. 131436), and p-4EBP1 (Cat. No. 75767) were purchased from Abcam (Cambridge, MA, USA). PARP-1 (Cat. No. 9542S) and SIRT1 (Cat. No. 9475 T) antibodies were purchased from Cell Signaling Technology (Shanghai, China). pADPr (PAR) (Cat. No. 56198) and AIF (Cat. No. 9416) antibodies were acquired from Santa Cruz Biotechnology (Beijing, China). The β-ACTIN (Cat. No. 21800) and LaminB1 (Cat. No. 40413) antibodies, in addition to secondary antibodies, were obtained from Signalway Technology (St. Louis, MO, USA). Hoechst/PI and EX527 (a SIRT1 inhibitor) were purchased from Beyotime Biotechnology (Shanghai, China). Rapamycin (an mTOR inhibitor) and 3-Aminobenzamide (3AB, a PARP-1 inhibitor) were obtained from Abmole (Beijing, China). All other reagents were acquired from Sigma-Aldrich (Shanghai, China).

### Cell culture

The mouse cone photoreceptor cell line 661 W was provided by Dr. Muayyad Al-Ubaidi (University of Oklahoma Health Science Center, Oklahoma, USA). Cell culture medium and all additives were purchased from the HyClone Company (Beijing, China). Cells were maintained in Dulbecco Modified Eagle Medium (DMEM) with 10% fetal bovine serum, 100 IU/mL streptomycin, and 100 IU/mL penicillin. Cells were cultured in a humid atmosphere at 37 °C with 95% air and 5% CO_2_. Trypsin digestion with 0.05% trypsin-EDTA was generally conducted on cells for 3–4 days, after which they were passaged at a 1:6 ratio.

### Experimental treatments of cells and lighting regimes

Light exposure experiments were performed as previously described [40]. Briefly, 661 W cells were cultured with normal DMEM medium in 6-well or 96-well plates for 24 h. The media were then replaced with sufficient fresh media following light exposure. To maintain consistent culture conditions, the light-exposed cells and control cells incubated in the dark were cultured in the same incubator. A standard 8-watt fluorescent strip light covered with a filter that confined the cells to exposure to the visible spectrum (400–800 nm) was placed 20 cm directly above the plates such that all cells received the same intensity of light exposure (1500 lx), as measured by a digital light meter (TES-1332A, Taipei, China). After the cells were exposed to visible light for 72 h, the experiment was terminated and cells were collected for further analysis. The dark control cell cultures were covered with card hoods that completely prevented light from entering. Cell media were changed every 2 days until the experiment was terminated. For the EX527 treatment conditions, the cells were pretreated with 150 μM EX527 / vehicle for 6 h before the light exposure procedure. The media were then replaced with fresh DMEM media and the cells were cultured under light/dark conditions for 72 h.

### PARP-1/mTOR/AIF knockdown with lentiviral-mediated short hairpin RNA (shRNA)

The lentivirus expressed short hairpin RNA (shRNA) targeting the PARP-1/mTOR/AIF gene was constructed by GeneCopoeia (Shanghai, China), with the lentivirus-mediated scrambled shRNA generated as a negative control. The interfering sequences specifically targeting the PARP-1/mTOR/AIF gene was as follows.

PARP-1 forward sequence:

5′-GATCCGGAGTACATTGTCTACGACATTTCAAGAGAATGTAGACAATGTACTCTTTTTTGGAATT-3′;

mTOR forward sequence:

5′-GATCCGGACACTTGGTTACAGGTTATATTCAAGAGATATAACCTGTAACCAAGTGTCTTTTTTGGAATT-3′;

AIF forward sequence:

5′-GATCCGCTCTTCAACATTCATGAAGATTCAAGAGATCTTCATGAATGTTGAAGAGTTTTTTGGAATT-3′.

The lentivirus particles were produced with a third-generation lentivirus packaging system. Briefly, three auxiliary plasmids (pRRE, pRSV-Rev, and pCMV-VSVG) and the core plasmid were transfected into human embryonic kidney 293 T cells (HEK293T) using the Lipo6000 transfection reagent (Beyotime Biotechnology, Shanghai, China). The viral particles were extracted 72 h after transfection, and lentivirus particles were further concentrated. Six hundred sixty one W cells were then infected with virus particles, and polybrene (Beyotime Biotechnology, Shanghai, China) was added to enhance infection efficiency. To avoid virulence, media were replaced with fresh complete DMEM media 24 h later. To obtain the cells that were consistently knocking down the target gene, cells were screened with hygromycin (Sangon Biotech, Shanghai, China) or puromycin (Beyotime Biotechnology, Shanghai, China) at an initial concentration of 8 μg/mL 2 days after infection of viral particles. Two weeks after screening, cells with stable PARP-1/mTOR/AIF knockdowns were obtained, while uninfected cells were killed at 48 h by hygromycin or puromycin treatment [39].

### AIF^GFP^ plasmid construction and transient transfection

Plasmids expressing AIF^GFP^ were purchased from Gene Copeia (Catalog No. EX-Mm06506-Lv122). Briefly, the plasmid (2.5 μg) and Lipo6000 (5 μl) were mixed with 125 μl of serum-free and antibiotic-free media for 5 min each. The mixtures were then combined and incubated for another 20 min, after which they were transferred into culture media with 661 W cells. After incubation for 6 h, the media were replaced with complete fresh media, and the cells were continuously cultured for 24 h. Cells transfected with AIF^GFP^ were then further prepared for light exposure experiments. The nuclear translocation of AIF was observed and photographed with a laser confocal microscope (Olympus FV100) 72 h after light exposure.

### Propidium iodide (PI)/Hoechst staining

Hoechst 33258 dye and propidium iodide (PI) were purchased from Beyotime Biotechnology (Shanghai, China). Briefly, cell nuclei were counter-stained with Hoechst 33258 dye (2 μg/mL) at 37 °C in the dark for 30 min, after which cells were stained with PI (5 μg/mL) under the same conditions for 10 min. The cells were subsequently observed and photographed using a fluorescence microscope (Olympus, Japan) and the photos were analyzed using the Image J software program (v1.51, NIH, USA). The percentage of cell death was then calculated using the following equation: PI positive cells/total cells × 100.

### Separation of cellular fractions

Cellular fractions were separated with a commercial assay kit (Beyotime Biotechnology, Shanghai, China), as previously described [41]. Cytoplasmic protein extraction by adding 200 μl of reagent A containing 1 nM Phenylmethanesulfonyl (PMSF) fluoride to every 20 μl aliquot of cell precipitates and incubating at 4 °C for 10 min. Next, 10 μl of reagent B was added and incubated at 4 °C for another 1 min. The cell and reagent mixtures were then vortexed and centrifuged at 12,000 g for 5 min, and the supernatant containing the cytoplasmic protein fraction was separated. To further purify the nuclear protein fraction, the precipitate was washed with PBS and centrifuged. Then, 50 μl of nucleoprotein extraction reagent containing 1 nM PMSF was added, and the mixture was vortexed and incubated at 4 °C for 30 min. The mixture was then centrifuged at 12,000 g at 4 °C for 10 min, followed by removal of the supernatant containing the nuclear protein fraction. The cytoplasmic and nuclear fraction samples were frozen at − 20 °C until immunoblotting analysis (Additional file 1: Supplementary Materials and Methods).

### Western blot analysis

Cells and retina samples were sonicated in protein lysate buffer, and a bicinchoninic acid assay was used to estimate protein contents. Briefly, an equal amount (20 μg) of cell lysate was dissolved in sample buffer, and samples were boiled for 3 min. Electrophoresis of sample solutions was then performed with 10% polyacrylamide gels containing 0.1% SDS. Proteins were then transferred to nitrocellulose membranes that were subsequently blocked with 5% non-fat dry milk in Tris-buffered saline with 0.1% Tween-20 (TBS-T) for 1 h at room temperature. The blots were then incubated for 3 h at room temperature with the primary antibodies, followed by incubation with the appropriate peroxidase-linked secondary antibodies. Signals were then developed using enhanced chemiluminescence and images were captured microscopically with a CCD camera (Tanon, Shanghai). Finally, densitometric analysis was performed using the Quantity One software program (Bio-Rad Laboratories).

### Animal experiments

All animal experiments were conducted in accordance with the Association for Research in Vision and Ophthalmology Statement for the Use of Animals in Ophthalmic and Vision Research. Eight-week old Male BALB/C57 mice were purchased from the Animal Center of Jilin University (Changchun). Mice were randomly divided into four groups with six individuals in each: control, Lt + vehicle, Lt + rapamycin, and Lt + 3AB. As previously described [39], the mice were fed in an animal room with a temperature between 21 °C and 23 °C and kept under a 12 h, 2.5 lx light/dark cycle. Next, 3AB (20 mg/kg body weight) or rapamycin (15 mg/kg body weight) dissolved in physiological saline containing 0.5% DMSO were intraperitoneally administered to mice for seven consecutive days. On the third day of administration, the mice were subjected to a previously described light exposure procedure [39]. Briefly, the pupils of the mice were dilated with 1% atropine eyedrops and the mice were continuously exposed to 7000 lx light for 12 h. After light exposure, the mice were kept in the animal room under a normal light/dark cycle. Retinal function was evaluated by electroretinography (ERG) 5 days later. After ERG evaluation, the mice were sacrificed by excessive injection with sodium pentobarbital (Beijing, China) and the eyeballs were immediately enucleated for morphological analysis and the retinas separated for Western blot analysis.

### Electroretinography (ERG)

Retinal function was evaluated using an electroretinogram (Metrovision, Perenchies, France). Dark-adapted (scotopic 0.01) and light-adapted (photopic 3.0 ERG) ERG were performed on all animals before the 3AB/rapamycin injections to establish baseline values. The ERGs were obtained 5 days after light exposure for the light exposure experimental group. All mice were dark-adapted 2 h before ERG analysis, and all preparations prior to measurement were conducted under dim red light. Mice were anesthetized via an intraperitoneal injection of sodium pentobarbital (60 mg/kg body weight) that was sufficient to maintain effective anesthesia for 45–60 min. The pupils were then dilated with a few drops of 1% tropicamide in saline (SINQI, Shenyang, China). Oxybuprocaine (Santen, Jiangsu, China) was then applied topically for corneal anesthesia, and carbomer (BAUSCH&LOMB, Shandong, China) was applied for corneal hydration. The animals were then placed on a heating pad that maintained their body temperatures at 35–36 °C throughout the experiment. The ground electrode was a needle inserted subcutaneously into the tail, and reference electrodes were placed subcutaneously in the lower jaw. The active recording electrodes were silver wires placed on the cornea. After setup under dim red light, another 10 min of dark adaptation was allowed before the commencement of measurements. ERG analyses were based on the amplitude measurements of the A- and B-waves.

### Retinal histology and retinal thickness measurements

The enucleated eyes were fixed in 4% paraformaldehyde (PFA) for 24 h at 4 °C, dehydrated with a serial gradient of ethanol concentrations, and then embedded in paraffin. The entire retina was sliced along the sagittal plane and the sections were stained with hematoxylin and eosin prior to photographing microscopically (Olympus, Japan). The thicknesses of the outer nuclear layers (ONLs) from the optic disc were measured at 500 μm intervals, as previously described [42]. The number of photoreceptor nuclei at a distance of 400–500 μm from the optic disc were quantified with the Image J software program (v1.51, NIH, USA) [43]. Three unrelated participants measured the thicknesses of the ONL and the number of photoreceptor nuclei to ensure the objectivity of the experimental data.

### Statistical analyses

Each experiment was repeated at least three times. Data are expressed as the means ± SEM. Differences between means were evaluated using one-way ANOVA tests followed by Bonferroni corrections using the GraphPad software program (statistical significance was set at *: *P* < 0.05, **: *P* < 0.01, ***: *P* < 0.001).

## Results

### mTOR/PARP-1 knockdown protects photoreceptors against light injury

**Fig. 1:**
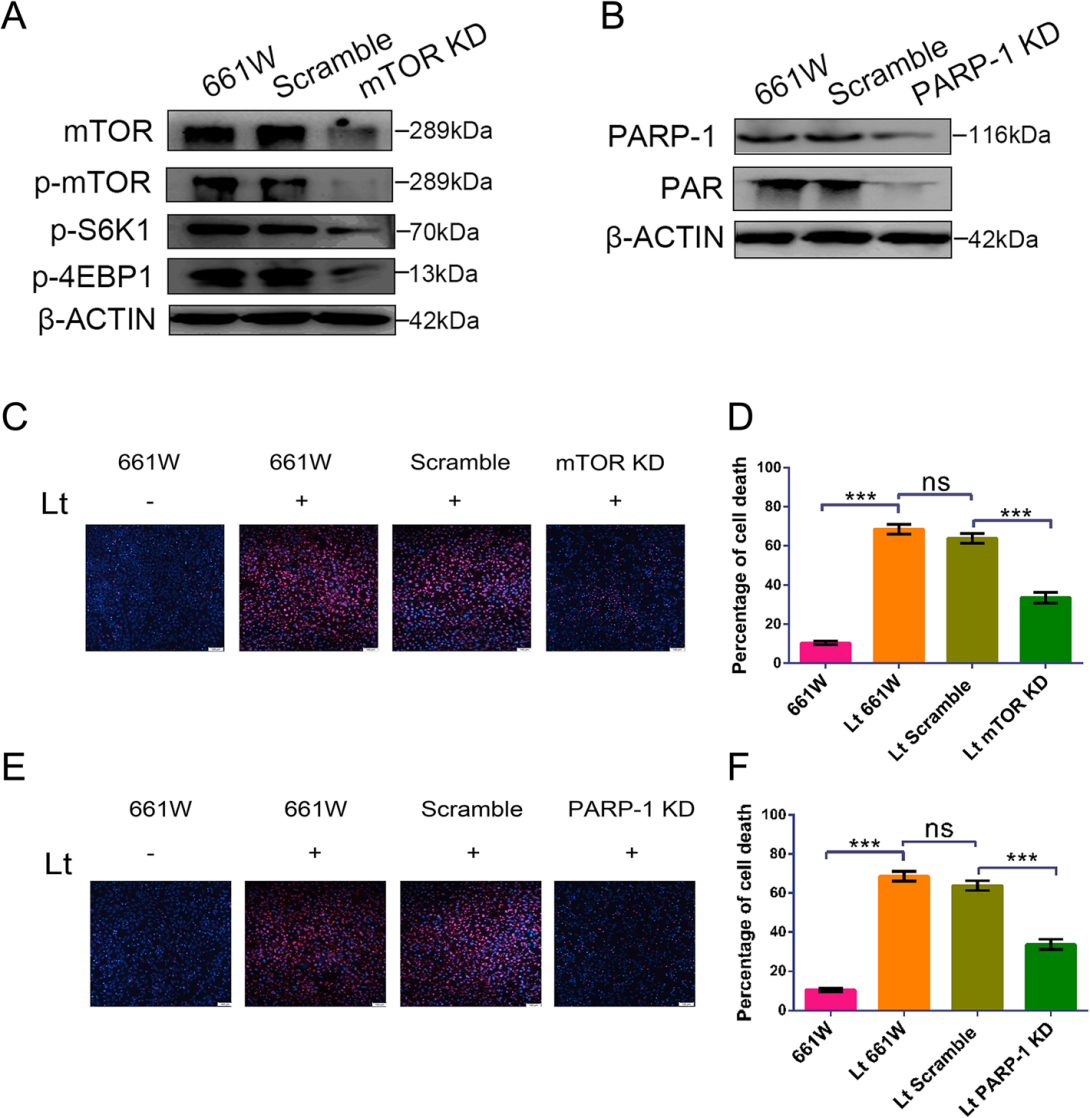
mTOR/PARP-1 knockdown protects photoreceptor cells against light injury. **a**, **b** 661 W cells were transfected with specific shRNAs targeting mTOR/PARP-1 genes, and cell lysates were analyzed by Western blot. β-ACTIN was used as an internal control. Scramble: cells were transfected with scrambled shRNA as a negative control; mTOR KD: cells with mTOR knockdown; PARP-1 KD: cells with PARP-1 knockdown. **c**, **e** Cells were exposed to 1500 lx light for 72 h and cell mortality was determined by PI/Hoechst staining. Scale bar = 100 μm. **d**, **f** Quantitative analysis of cell mortality. Lt: light exposure for 72 h. All experiments were repeated in triplicate, and the results are shown as the means ± SEM (*n* = 3, ***: *P* < 0.001, ns: not statistically significant)

To assess the roles of mTOR and PARP-1 in the mechanism of light-induced death, 661 W cells with stable mTOR/PARP-1 knockdowns were screened using the lentivirus-mediated shRNA methodology combined with hygromycin or puromycin treatment. The expression of both mTOR and phosphorylated mTOR (p-mTOR) in mTOR-KD cells was remarkably reduced compared with that in scramble or 661 W cells (Fig. 1a). Consistent with this, downstream factors of the mTOR signal, phosphorylated 4EBP1 (p-4EBP1), and phosphorylated S6K1 (p-S6K1) also exhibited clear down-regulation. Further, the expression of PARP-1 and its product PAR was markedly decreased in PARP-1-KD cells compared with that in scramble or 661 W cells (Fig. 1b). These results suggest that cell lines with stable mTOR/PARP-1 knockdowns were successfully established. The role of mTOR-KD or PARP-1-KD in light exposure-induced damage was then further evaluated. mTOR-KD or PARP-1-KD cells were exposed to 1500 lx visible light for 72 h and cell mortality was quantitatively determined by Hoechst/PI staining. After 72 h of 1500 lx light exposure, nearly 68.53% ± 2.53 of the 661 W cells and 63.85% ± 2.49 of the scramble cells had died (Fig. 1c-f), representing significantly greater dead cells than the dark control treatment. However, knockdown of mTOR/PARP-1 corresponded with remarkable protection against light injury, with significantly reduced cellular mortalities of 32.68% ± 2.79 in mTOR-KD cells and 33.73% ± 2.67 in PARP-KD cells. Thus, these findings indicate that mTOR and PARP-1 play important roles in the mechanism of visible light-induced death in photoreceptor cells.

### PARP-1 and mTOR are activated and interact in light exposure-induced damage

**Fig. 2:**
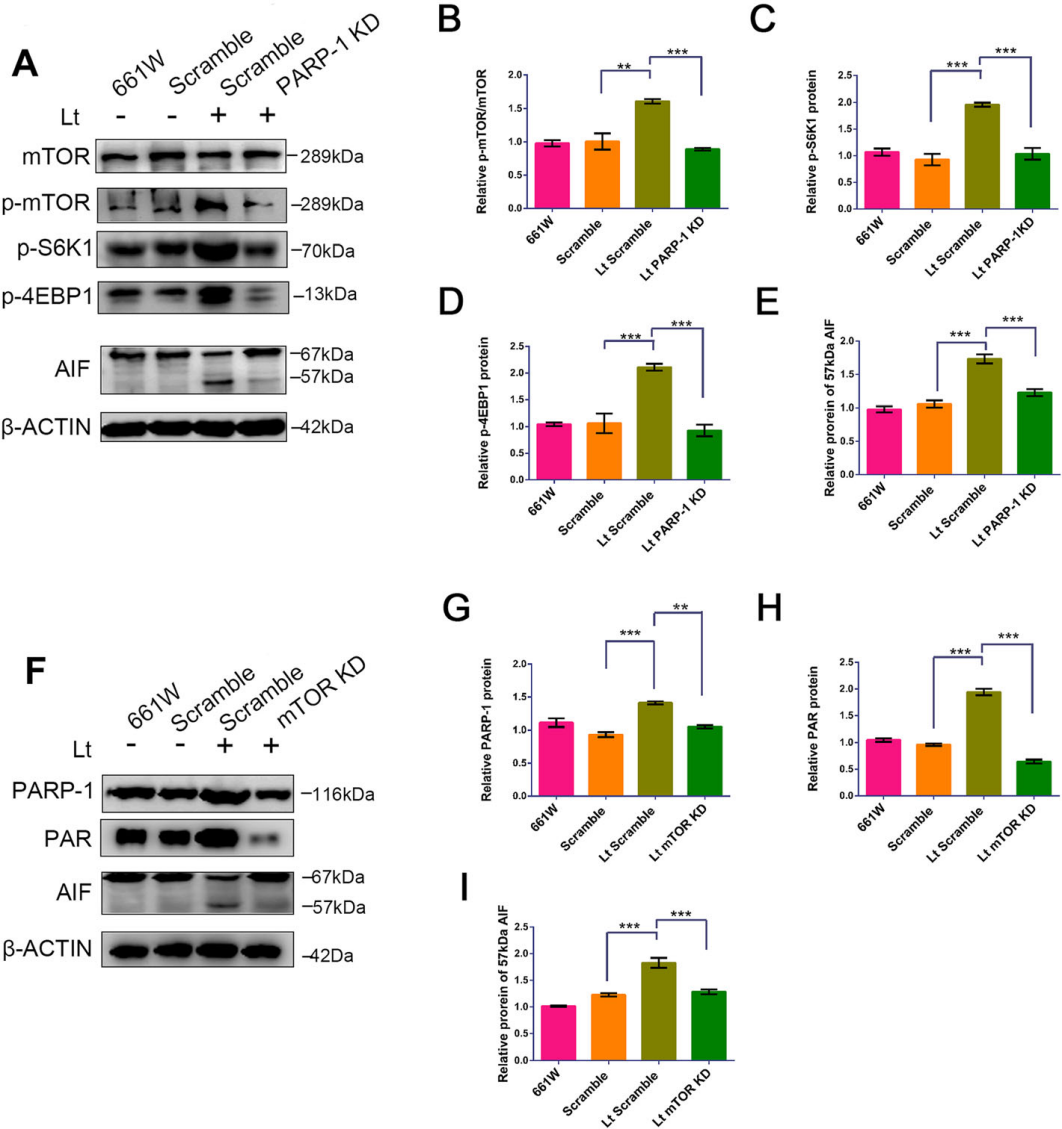
PARP-1 and mTOR are activated and interact during light exposure-induced damage. **a**, **f** Cells were cultured under light/dark conditions for 72 h, followed by cell lysing and analysis with Western blot. β-ACTIN was used as an internal control. Scramble: cells were transfected with scrambled shRNA as a negative control; mTOR KD: mTOR knockdown; PARP-1 KD: PARP-1 knockdown; Lt: 1500 lx light exposure for 72 h. **b–e**, **g–i** Quantitative analysis of target protein levels related to β-ACTIN. All experiments were repeated in triplicate, and the results are shown as the means ± SEM (**: *P* < 0.01, ***: *P* < 0.001)

After visible light exposure of 1500 lx for 72 h, p-mTOR levels were significantly up-regulated (Fig. 2a-e), and the p-mTOR/mTOR ratio markedly increased in the scramble cells compared to dark control cells. Moreover, the levels of both p-S6K1 and p-4EBP1, which are downstream factors of the mTOR signal, were also significantly up-regulated. However, PARP-1 knockdown significantly suppressed the activation of the mTOR signal caused by light exposure, leading to a significant reduction in the p-mTOR/mTOR ratio in addition to levels of p-S6K1 and p-4EBP1 compared to scramble cells. Similarly, 72 h of light exposure resulted in significant activation of the PARP-1 signal, as indicated by a remarkable increase in the levels of both PARP-1 and its product PAR in scramble cells compared to dark control cells (Fig. 2f-i). In addition, the downstream factor in parthanatos, the 57 kDa active form of AIF, was detected in light-exposed cells. However, mTOR knockdown abolished the activation of the PARP-1 signal, leading to a significant reduction in the levels of both PARP-1 and its PAR product relative to scramble cells. Interestingly, AIF activation was also suppressed by mTOR knockdown, as indicated by a reduced 57 kDa AIF form in mTOR-KD cells, even under light exposure conditions. Taken together, these results suggest that the PARP-1 and mTOR signals are both activated by light exposure, and there is crosstalk between the signals.

### Visible light exposure leads to parthanatos in photoreceptor cells

**Fig. 3:**
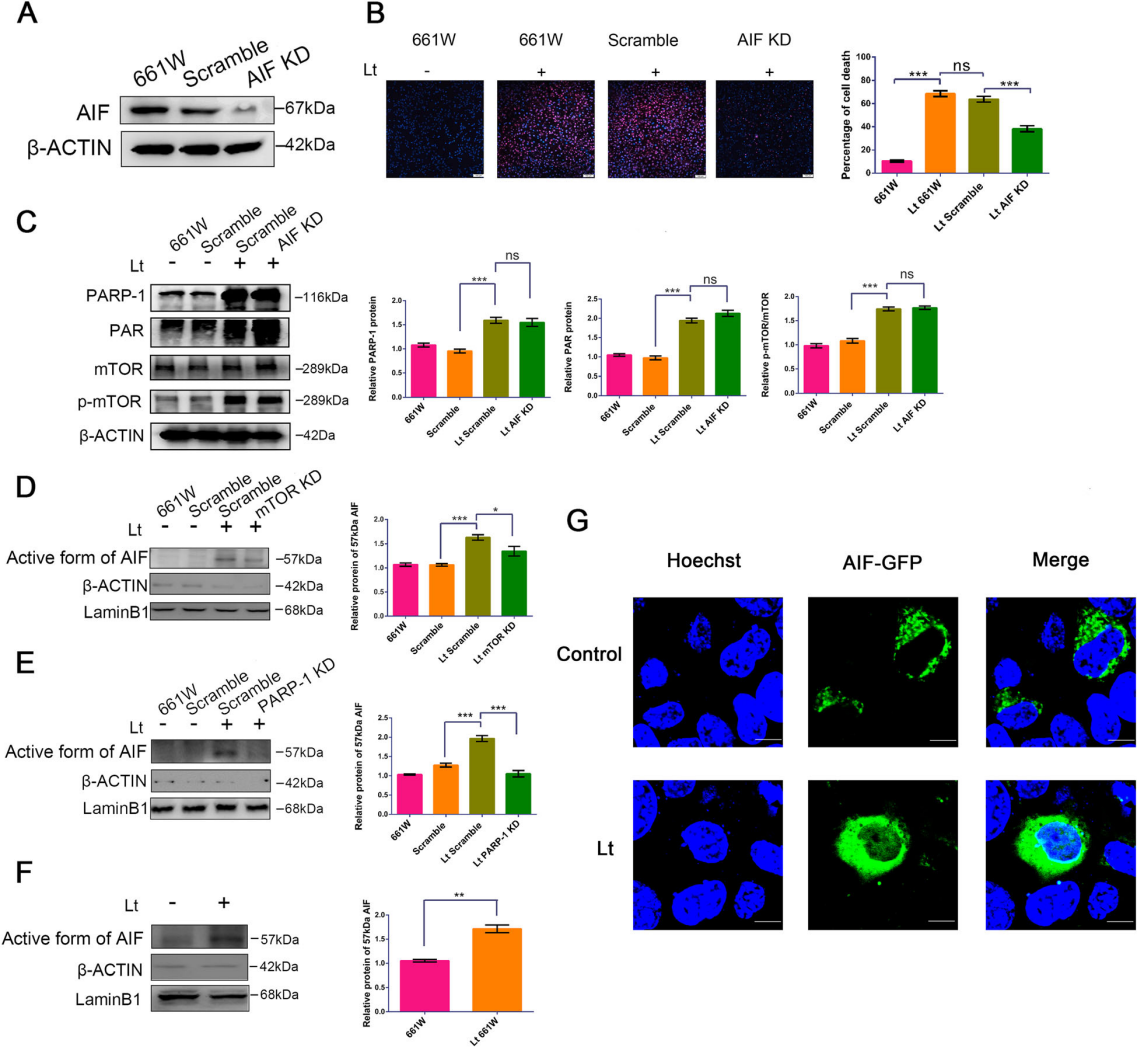
AIF knockdown protects 661 W cells against light injury, but PARP-1 and mTOR function as upstream factors. **a** 661 W cells were transfected with specific shRNAs targeting the AIF gene, cells were lysed, and lysates were analyzed by Western blot. β-ACTIN was used as an internal control. Scramble: cells transfected with scrambled shRNA as a negative control; AIF KD: cells with AIF knockdown; Lt: 1500 lx light exposure for 72 h. **b** Cell mortality was quantified by PI/Hoechst staining. Scale bar = 100 μm. **c** Cells were cultured under light/dark conditions for 72 h, followed by cell lysis, and analysis of lysates with Western blot. β-ACTIN was used as an internal control. **d-f** Cells were cultured under light/dark conditions for 72 h, and the nuclear fractions were separated and analyzed by Western blot. LaminB1 and β-ACTIN were used as nuclear and cytosol fraction controls, respectively. The active form of 57 kDa AIF was quantitatively analyzed relative to LaminB1. mTOR KD: mTOR knockdown; PARP-1 KD: PARP-1 knockdown. All experiments were repeated in triplicate, and the results are shown as the means ± SEM (*: *P* < 0.05, **: *P* < 0.01, ***: *P* < 0.001, ns: no statistical significance). **g** 661 W cells expressing AIF-GFP were cultured under light/dark conditions for 72 h and images were captured with a confocal laser microscope. The nuclei were counter-stained with Hoechst staining. Scale bar = 20 μm

During parthanatos, over-produced PAR results in the permeabilization of the outer membrane of mitochondria, thereby promoting the release of the 57 kDa active AIF and its subsequent nuclear translocation. To confirm the cell death mechanism, the role of AIF as the executor in parthanatos was further evaluated in light exposure-induced cell death. We first screened 661 W cells with stable AIF knockdowns. The expression of AIF was efficiently suppressed by lentivirus-mediated specific shRNA relative to scramble cells (Fig. 3a). More importantly, PI/Hoechst staining demonstrated that AIF knockdown significantly reduced cell death prevalence when compared to scramble cells after exposure to 1500 lx light for 72 h (38.23% ± 2.65 in AIF-KD cells vs 63.85% ± 2.49 in scrambled cells) (Fig. 3b). These results indicate that AIF plays an important role in light-induced death of photoreceptors. We therefore verified the nuclear translocation of AIF caused by light exposure with a cellular fraction assay. The active form of AIF was clearly detected in the nuclear fraction of scramble cells at 72 h after 1500 lx light exposure (Fig. 3d, e), and significant up-regulation was observed in those cells compared to dark-incubated control cells. Similar observations were made for 661 W cells (Fig. 3f). Moreover, mTOR and PARP-1 knockdowns were both able to significantly suppress the up-regulation of the 57 kDa AIF due to light exposure in the nuclear fraction when compared to scramble cells. However, AIF knockdown did not influence the levels of PARP-1 and mTOR signals (Fig. 3c), indicating that both PARP-1 and mTOR are upstream factors of AIF. However, the levels of 57 kDa AIF in the nuclear fraction were more significantly reduced by PARP-1 knockdown rather than mTOR knockdown (Fig. 3d, e), suggesting that PARP-1 may be a direct factor in the regulation of AIF activation, while mTOR could be an indirect factor. We also evaluated the nuclear translocation of AIF using a fusion protein methodology with a plasmid expressing AIF^GFP^. The AIF-GFP was primarily located in the cytoplasm under dark-incubation treatments, resulting in green-colored cytoplasms (Fig. 3g), while AIF clearly translocated into nuclei after exposure to 1500 lx light for 72 h, as indicated by light-blue fluorescence in Hoechst-stained nuclei. Taken together, these results suggest that visible light exposure leads to parthanatos-like death in photoreceptors and that mTOR and PARP-1 may function as upstream factors of AIF to regulate the death process cascade.

### PARP-1 and mTOR interact via SIRT1

**Fig. 4:**
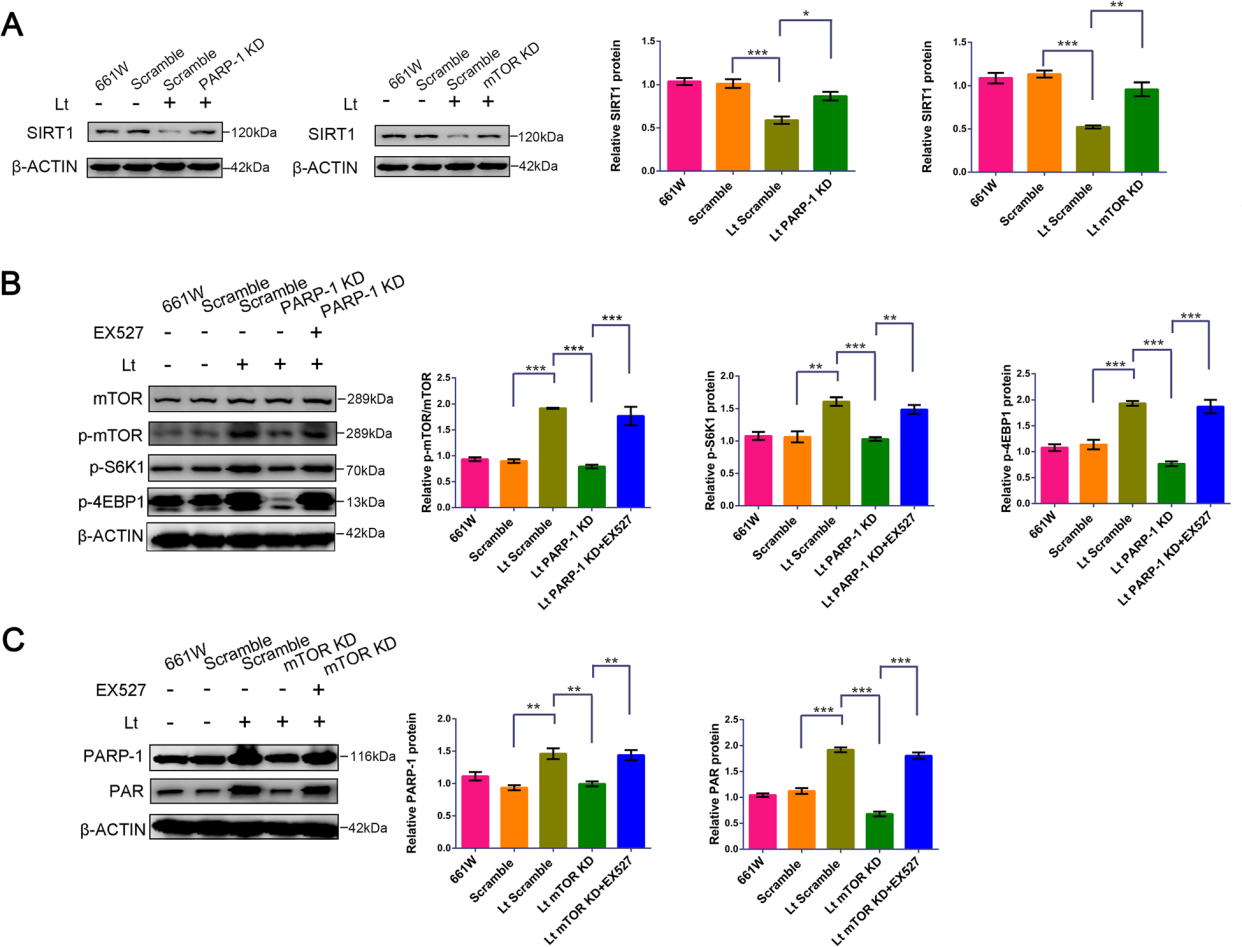
SIRT1 inhibition blocks crosstalk between mTOR and PARP-1 signals under light exposure conditions. **a-c** Cell lysate samples were analyzed by Western blot with β-ACTIN used as an internal control. Scramble: cells transfected with scrambled shRNA were used as a negative control; mTOR KD: mTOR knockdown; PARP-1 KD: PARP-1 knockdown; Lt: 1500 lx light exposure for 72 h. EX527: a SIRT1 inhibitor. The target proteins levels were quantitatively analyzed relative to β-ACTIN. All experiments were repeated in triplicate, and the results are shown as the means ± SEM (*: *P* < 0.05, **: *P* < 0.01, ***: *P* < 0.001)

**Fig. 5:**
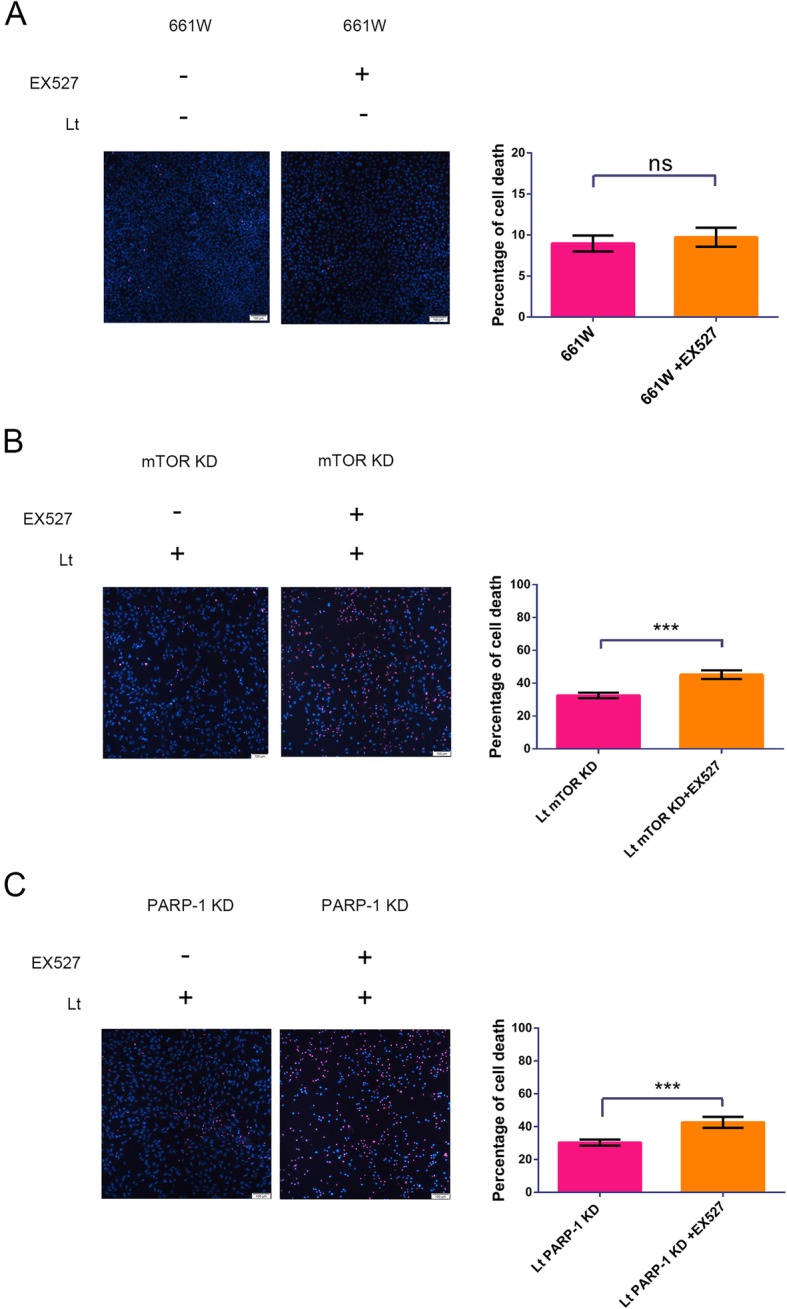
SIRT1 inhibition inhibits the protection induced by mTOR/PARP-1 knockdown against light injury. **a** 661 W cells were pretreated with 150 μM EX527/vehicle for 6 h and cultured with fresh medium under normal conditions for 72 h. Cell mortality was then quantified by PI/Hoechst staining. **b**, **c** Cells with mTOR/PARP-1 knockdowns were pretreated with 150 μM EX527/vehicle for 6 h and exposed to 1500 lx light while culturing with fresh medium for 72 h. Cell mortality was quantified by PI/Hoechst staining. mTOR KD: mTOR knockdown; PARP-1 KD: PARP-1 knockdown; Lt: light exposure for 72 h; EX527: SIRT1 inhibitor. Scale bar = 100 μm. All experiments were repeated in triplicate, and the results are shown as the means ± SEM (**: *P* < 0.01, ns: not statistically significant)

PARP-1 and SIRT1 have the potential to compete for the same NAD^+^ substrate. However, SIRT1 is an NAD^+^-dependent protein deacetylase that limits the availability of NAD^+^ and could be a key link between PARP-1 and mTOR. To assess the role of SIRT1 in PARP-1 and mTOR crosstalk, we interrupted the PARP-1 and mTOR signal loop with a specific inhibitor of SIRT1. Light exposure led to significant suppression of SIRT1 compared to dark control cells (Fig. 4a), while PARP-1/mTOR knockdown attenuated the suppression of SIRT1. Consistent with Western blot analyses (Fig. 4a), mRNA levels of SIRT1 remarkably decreased in light-damaged 661 W cells compared with control cells (Additional file 2: Figure S1). Further, PARP-1 knockdown significantly abolished the activation of the mTOR signal caused by light exposure (Fig. 4b), resulting in reduced p-mTOR/mTOR values and expression of p-S6K1 and p-4EBP1 compared to scramble cells. Treatment with 150 μM of EX527 remarkably mitigated the PARP-1 knockdown-induced suppression of the mTOR signal, with increased levels again in p-mTOR/p-S6K1/p-4EBP1 after light exposure compared to untreated PARP-1 KD cells. Similarly, mTOR knockdown resulted in the inactivation of PARP-1 after light exposure (Fig. 4c), leading to significantly reduced PARP-1 and PAR levels compared to scramble cells. However, treatment with 150 μM of EX527 also decreased mTOR knockdown-induced inhibition of the PARP-1 signal, as indicated by re-upregulation of PARP-1 and PAR levels compared to untreated mTOR KD cells. Furthermore, either PARP-1 or mTOR knockdown caused up-regulation of SIRT1 activity, while treatment with 150 μM EX527 significantly reduced the activity of SIRT1 compared with the vehicle group (Additional file 2: Figure S2). More importantly, treatment with 150 μM EX527 significantly lessened the protection caused by mTOR knockdown following light exposure, as evinced by increased cell mortality from 32.5% ± 1.66 to 45.15% ± 2.65 in the EX527 treatment. Similarly, PARP-1 knockdown-induced protection after light exposure was also lessened by treatment with 150 μM EX527, as indicated by an increase in cell mortality from 30.3% ± 1.83 to 42.62% ± 3.32 in the EX527 treatment. Under normal cell culture conditions, treatment with EX527 had no effect on the rate of cell death (Fig. 5). Thus, these findings suggest that SIRT1 is an important intermediate factor involved in crosstalk between the PARP-1 and mTOR signals.

### The PARP-1 and mTOR pathways are activated and interact in light-injured retina

**Fig. 6:**
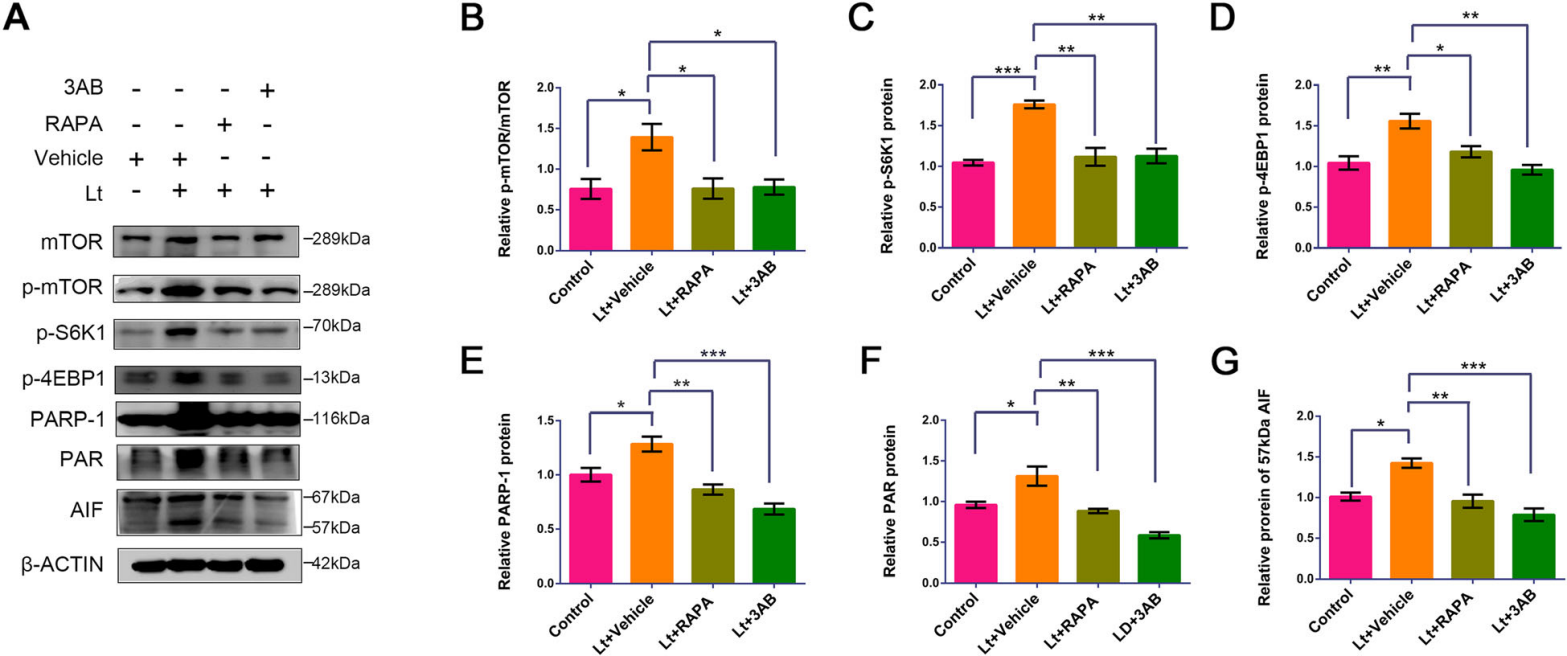
PARP/mTOR inhibitors attenuate the light-induced activation of PARP-1/mTOR signals in mice retina. Mice were intraperitoneally injected with 20 mg/kg 3AB or 15 mg/kg rapamycin for 7 days following 7000 lx light exposure for 12 h. **a** The mice were sacrificed 5 days after light exposure, and retinal samples were analyzed by Western blot. β-ACTIN was used as an internal control. RAPA: rapamycin, an mTOR inhibitor; 3AB: a PARP-1 inhibitor; Lt: 7000 lx light exposure for 12 h. **b–g** Quantitative analysis of target protein levels related to β-ACTIN. All experiments were repeated in triplicate, and the results are shown as the means ± SEM (*: *P* < 0.05, **: *P* < 0.01, ***: *P* < 0.001)

To explore the roles of PARP-1 and mTOR in the mechanisms underlying retina light injury in vivo, a light-damaged mouse model was established and light-injured retinas were prepared. After exposure to 7000 lx light for 12 h, the mTOR signal was significantly activated in the retina (Fig. 6), with an obviously increased p-mTOR/mTOR ratio, in addition to increased p-4EBP1 and p-S6K1 expression in the light-treated group compared to the control group. As the critical factors in parthanatos, PARP-1 and PAR were also activated following light exposure with prominent increases in protein levels compared to controls. Moreover, as the downstream factor of PARP-1 in parthanatos, the 57 kDa active form of AIF was detected in light-treated retinas. The interactions between PARP-1 and the mTOR signal were further investigated in vivo using their inhibitors. The intraperitoneal administration of the PARP-1 inhibitor 3AB suppressed activation of the mTOR signal caused by light exposure, leading to a significant reduction in the levels of p-mTOR/mTOR ratios, as well as the expression of p-4EBP1 and p-S6K1 (Fig. 6). Moreover, light exposure-induced activation of AIF was remarkably suppressed by PARP-1 inhibition. Similarly, intraperitoneal injection of the mTOR inhibitor, rapamycin, resulted in significant down-regulation of PARP-1 and PAR compared to levels in vehicle-treated retinas following light exposure. The inhibition of mTOR also led to the inactivation of AIF in the light-exposed retinas, as noted by significantly reduced levels of the 57 kDa AIF. Thus, these results suggest that light exposure induces parthanatos-like injury in the retina and that mTOR may interact with PARP-1 to regulate parthanatos progression.

### mTOR/PARP-1 inhibition protects the retina against light damage

**Fig. 7:**
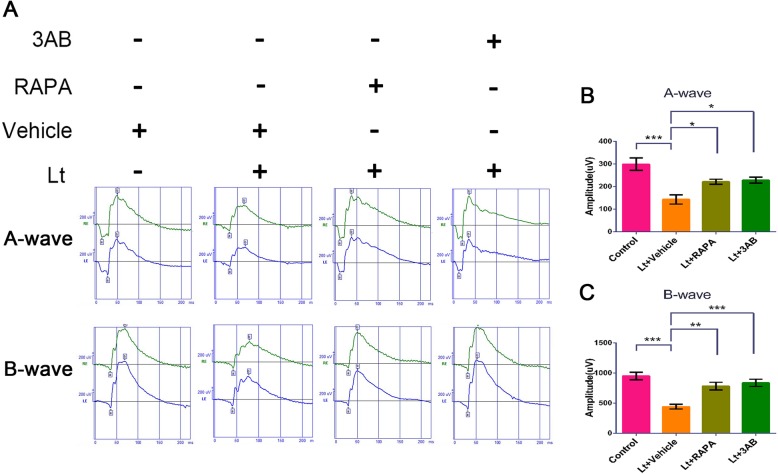
PARP-1/mTOR inhibition attenuates retina light injury. The mice were intraperitoneally injected with 20 mg/kg 3AB or 15 mg/kg rapamycin for 7 days following 7000 lx light exposure for 12 h. Retinal function was evaluated with ERG analyses 5 days after the light exposure. **a** ERG waves. **b**, **c** Quantification of the A- and B-wave values. All experiments were repeated in triplicate, and the results are shown as the means ± SEM (*: *P* < 0.05, **: *P* < 0.01, ***: *P* < 0.001)

**Fig. 8:**
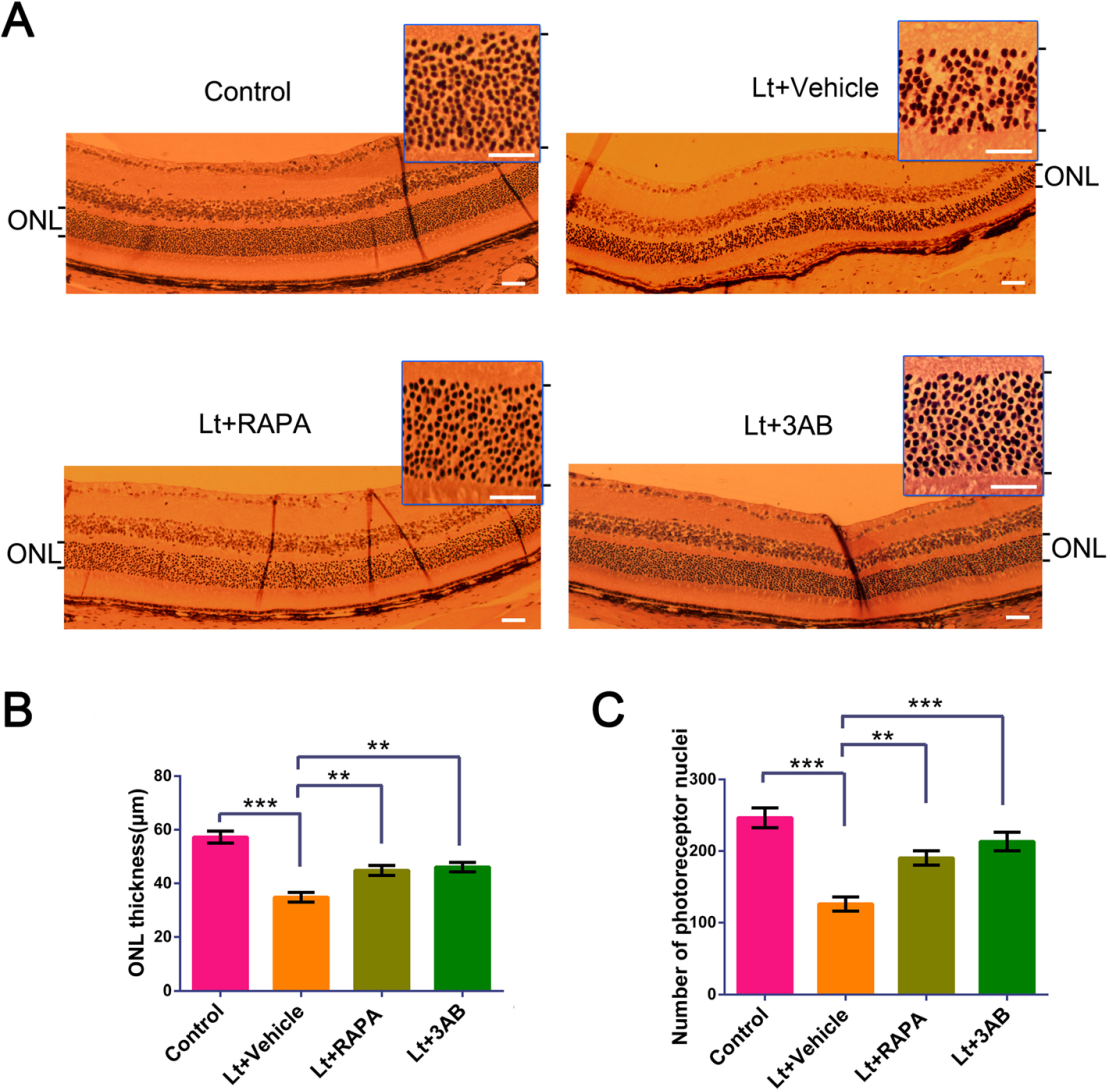
PARP-1/mTOR inhibition attenuates light-induced retina ONL thinning. Mice were intraperitoneally injected with 20 mg/kg 3AB or 15 mg/kg rapamycin for 7 days following 7000 lx light exposure for 12 h. Mice were sacrificed and retinas were sectioned for histopathological analysis. **a** histopathological analysis of mice retina. Scale bar = 20 μm. **b** Quantification of ONL thickness. **c** Quantification of photoreceptor nuclei. All experiments were repeated in triplicate, and the results are shown as the means ± SEM (**: *P* < 0.01, ***: *P* < 0.001)

The neuroprotective effects of mTOR/PARP-1 inhibition in light injured retinas was further evaluated using full retinal ERG analyses. Exposure to 7000 lx light for 12 h led to severe damage to retinal function (Fig. 7), as noted by a significant reduction in A- and B-wave values in ERG. However, intraperitoneal administration of 3AB (20 mg/kg) or rapamycin (15 mg/kg) significantly attenuated A- and B-wave amplitude decreases relative to the vehicle light-treated group. To further evaluate the structurally protective effects of the above, the thicknesses of the outer nuclear layers (ONLs) were evaluated with H&E staining. ONL thickness in the retinas from vehicle-treated mice following light exposure was significantly reduced compared to control mice (Fig. 8). Moreover, H&E staining revealed that the entire retina structure was disordered, with significant irregular arrangements in the ONL. Further, quantitative analysis revealed that the number of photoreceptor nuclei was significantly reduced and that the tight packing of photoreceptor nuclei was disrupted and replaced by loose and disordered nuclei structures. However, treatment with 3AB (20 mg/kg) or rapamycin (15 mg/kg) significantly mitigated light-induced damage to the structure of the retina, as indicated by increased ONL thicknesses and abundances of photoreceptor nuclei compared to the vehicle light-treated group. Taken together, these results suggest that PARP-1/mTOR inhibition provides remarkable protection of retinas against light injury.

## Discussion

PARP-1 is a nuclease and critically functions to sense DNA damage and maintain nuclear DNA homeostasis [44]. As PARP-1 detects DNA breaks, it binds to the DNA and synthesizes PAR polymers that act as signals to activate other DNA-repairing enzymes [45]. Severe damage to DNA can lead to increases of over 500-fold in PARP-1 activity to synthesize a large amount of PAR polymers via oxidation of NAD^+^ and considerable ATP consumption [46]. However, the over-activation of PARP-1 can lead to parthanatos that is also known as PARP-1-dependent cell death [24]. Parthanatos is characterized by the accumulation of PAR, nuclear translocation of AIF from the mitochondria, and is involved in various retinal degeneration diseases. Donovan et al. found that the levels of intracellular calcium, superoxides, and mitochondrial membrane depolarization increased in BALB/C mice that were exposed to cool white fluorescent light at a luminescence of 5000 lx for 2 h, although caspase-3 was not activated [47]. Further, Paquet-Durand et al. found that the death of photoreceptor cells in RP rd1 mice was closely related to AIF nuclear translocation and oxidative DNA damage [20]. Accordingly, parthanatos might play an important role in the progression of retinal degeneration. In our previous studies, we found that caspase activation was not necessary during light-induced death of photoreceptor cells since Z-VAD-fmk, a pancaspase inhibitor, failed to protect photoreceptor cells from light injury. Concomitantly, PARP-1 inhibitors imparted protection, suggesting that light exposure leads to caspase-independent cell death [39]. In the current study, we further demonstrated that light exposure caused AIF activation and translocation of active AIF into the nucleus. In addition, the knockdown of PARP-1 clearly blocked the nuclear translocation of AIF. Both PARP-1 and AIF knockdown protected photoreceptor cells against light damage, significantly reducing cell mortality. Thus, we conclude that light-induced death in photoreceptors occurs via the parthanatos pathway that is PARP- and AIF-dependent. Consistently, we also observed the activation of AIF in vivo in the light-damaged retina of mice, while the PARP-1 inhibitor was able to mitigate the activation of AIF and significantly protect the retina against light injury.

Moreover, we found that light exposure caused marked activation of mTOR signals in photoreceptor cells. mTOR is a serine/threonine protein kinase that integrates a number of cellular pathways involving transcription, maturation, proliferation, and survival. mTOR activity is modulated through phosphorylation of its specific residues in response to alteration of nutritional status, mitogens, and other stimuli [27, 28, 48]. Increasing evidence indicates that the mTOR signal is involved in a variety of neurodegenerative diseases ranging from those affecting the central nervous system to eyes. However, the role of mTOR in neuroprotection is unique in that it can have both positive and negative effects. In Parkinson’s disease, mTOR inhibition may provide cytoprotection for post-mitotic neurons that attempt to enter the cell cycle [49]. Rapamycin offers neuronal protection in Parkinson’s disease models that is believed to function through the preservation of phosphorylation of Protein kinase B (Akt) at Thr308, thereby promoting cell survival [50]. Conversely, other studies have shown that mTOR activation protects degenerative neurons. In Alzheimer’s disease, rapamycin treatment may exacerbate amyloid toxicity [51]. Further, mTOR and p70S6K1 pathway activation can protect inflammatory cells responsible for Aβ sequestration, microglia, from the toxic effects of Aβ exposure [52]. Additionally, mTOR is important for axonal regeneration in glaucoma and traumatic optic neuropathy wherein the deletion of the Phosphatase and Tensin Homolog (PTEN), a negative regulator of mTOR, promotes robust retinal ganglion cell (RGC) axon regeneration within the injured optic projection [53]. In the current study, mTOR inhibition played a protective role in light-damaged photoreceptors both in vitro and in vivo. When mTOR was knocked down, we observed a remarkable decrease in 661 W cell mortality due to light exposure. Further, rapamycin treatment significantly mitigated light-induced injury in the mouse retina, both functionally and structurally.

Both PARP-1 and mTOR suppression are able to protect photoreceptors against light damage, and thus the potential crosstalk between the two signals and possible intermediate factors were further investigated. Knockdown of PARP-1 reduced the ratio of p-mTOR/mTOR and the levels of p-S6K1 and p-4EBP1. Similarly, mTOR knockdown caused obvious reductions in the levels of PARP-1 and PAR under light exposure conditions. These results suggest that there is indeed a close interaction between PARP-1 and mTOR signals and that the inhibition of PARP-1 or mTOR may reciprocally suppress the light-induced activation of the signal, although there is no upstream or downstream relationship between the two signals. To further investigate the crosstalk between PARP-1 and mTOR signals, we focused on SIRT1, which is a key enzyme that regulates the cellular NAD^+^ pool and could function as an intermediate factor connecting both the PARP-1 and mTOR signals.

Sirtuins are a family of NAD^+^-dependent protein deacetylases with key metabolic roles [54]. In addition, PARP-1 is an NAD^+^-dependent enzyme that potentially competes with restricted NAD^+^ substrates. PARP-1 utilizes NAD^+^ as a substrate to transfer ADP-ribose to the receptor protein during DNA break repairs [55, 56]. However, over-activation of PARP-1 results in almost complete depletion of NAD^+^ and ATP pools, which may greatly influence SIRT1 activity [57–59]. This observation led to the hypothesis that sirtuin and PARP activities might compete for a common NAD^+^ pool, as proposed by Zhang et al. [60]. In addition, the intracellular levels of NAD^+^ may also regulate both the expression and activity of SIRT1 through substrate availability. Jadeja et al. reported that the highly specific nicotinamide phosphoribosyltransferase (NAMPT) inhibitor, FK866, led to a dose-dependent decline in NAD^+^ content within cultured RPE cells, while the expression and activity of SIRT1 was also reduced in a dose-dependent manner [61]. In this study, light exposure led to severe photooxidative stress in 661 W cells. The excessive ROS could penetrate nuclear membranes, resulting in DNA breaks. PARP-1 acts as a nuclear enzyme responding to DNA damage by producing a large number of PAR-polymers while using NAD^+^ as a substrate. Overproduction of PAR-polymers may result in decreased NAD^+^ levels or NAD^+^ deficiencies that further suppresses the expression of SIRT1. Accordingly, we observed increased levels of PARP-1, but decreased levels of SIRT1, in light-damaged 661 W cells. In contrast, PARP-1 knockdown suppressed the excessive generation of PAR and abrogated NAD^+^ depletion in 661 W cells after light exposure, thereby resulting in recovery of SIRT1 levels and its associated deacetylase activity. These results are consistent with those of Bai et al. that reported PARP-1 transcription depletion increases NAD^+^ content and SIRT1 activity in the brown adipose tissues and muscles of PARP-1^−/−^ mice. In addition, the pharmacologic inhibition of PARP in vitro and in vivo causes increased NAD^+^ levels and SIRT1 activities [62].

mTOR signaling is a primary regulator of cellular metabolism and thus may also significantly influence cellular NAD^+^ contents, thereby indirectly regulating the expression and activity of SIRT1. In the present study, mTOR knockdown led to a re-increase of SIRT1 levels and activities in 661 W cells after light exposure, consistent with previous studies. Zhang et al. reported that rapamycin treatment could significantly recover cellular NAD^+^/NADH levels and increase the expression and activity of SIRT1 in order to delay the senescence of high glucose-inducing mesangial cells [63]. In addition, Back et al. demonstrated that mTOR activation caused phosphorylation of SIRT1 at the serine47 site, resulting in the inhibition of SIRT1 deacetylase activity in human squamous cell carcinoma (SCC) cells, although the pharmacologic and genetic inhibition of mTOR restored SIRT1 deacetylase activity [64].

To further understand the connection among PARP-1, SIRT1, and mTOR, we disrupted the signal loop by using a specific inhibitor of SIRT1, EX527. Treatment with EX527 remarkably abrogated the PARP-1 knockdown-induced decrease of p-mTOR/mTOR levels, while re-increasing p-mTOR/ mTOR levels after light exposure. Similarly, PARP-1 signal suppression from mTOR knockdown was also significantly abolished by EX527 treatment, resulting in increased PARP-1 and PAR levels. Additionally, the protection afforded by PARP-1 and mTOR knockdown against light injury were markedly attenuated by EX527 treatment. Consequently, we concluded that SIRT1 is an important intermediate factor in the crosstalk of PARP-1 and mTOR and negatively regulates their signals. The negative regulation of SIRT1 on PARP-1 and mTOR signals has been previously reported. Rajamohan et al. demonstrated that both overexpression of SIRT1 and activation of SIRT1 with resveratrol (RSV), a SIRT1 agonist, led to the deacetylation of PARP-1 in rat cardiomyocytes [58]. Further, Cantó et al. demonstrated that SIRT1-mediated deacetylation blocks the catalytic activity of PARP-1 [65]. In addition, Ghosh et al. showed that SIRT1 knockout caused elevated mTOR activity in HeLa cells and mouse mouse embryonic fibroblasts (MEFs). Moreover, Ghosh et al. observed that activating SIRT1 with RSV led to the inhibition of mTOR signals in response to specific stimuli, such as hormones or low energy availability. More importantly, the SIRT1 activator RSV was unable to inhibit S6K1 phosphorylation in the absence of Tuberous sclerosis complex 2 (TSC2), indicating that the inhibition of mTORC1 activity by SIRT1 is TSC2- dependent. It is thus possible that TSC2 may be acetylated in response to stress or growth conditions and is regulated by the deacetylase activity of SIRT1 [66].

## Conclusions

These results demonstrate for the first time that light exposure leads to parthanatos-like death in photoreceptors and that mTOR may regulate PARP-1-dependent cell death via SIRT1. Parthanatos-like death of photoreceptors due to light exposure may be initiated by photo-oxidative stress. Excessive ROS may penetrate the nuclear membrane, resulting in DNA breaks that further lead to the over-activation of PARP-1. To repair damaged nuclear DNA, PARP-1 produces a large amount of PAR polymers accompanied by the massive consumption of cellular NAD^+^ and ATP. PAR polymers result in increased mitochondrial permeability of the outer membrane and nuclear translocation of AIF, which further leads to large-scale fragmentation of DNA and cell death. However, further investigation is needed to determine how light exposure activates mTOR and the close connections among PARP-1, SIRT1, and mTOR signals require further elucidation. Overall, the results of this study provide new insight into the molecular mechanisms underlying photoreceptor/retina light injury. Importantly, these insights will facilitate the development of promising neuroprotective strategies for future clinical treatment of light injury-related retinal degeneration diseases.

## Supplementary information

**Additional file 1.** Supplementary Materials and Methods

**Additional file 2:** Supplementary Figure Legends. **Figure S1.** SIRT1 mRNA levels decreased in light-damaged 661 W cells compared to control cells. Quantitative real-time PCR analysis of SIRT1 mRNA expression. β-ACTIN was used as an endogenous control. SIRT1 expression levels were normalized to the mean expression levels of β-ACTIN. Lt: 1500 lx light exposure for 72 h. All experiments were repeated in triplicate and the results are shown as the means ± SEM (***: *P* < 0.001). **Figure S2.** PARP-1 / mTOR knockdown caused up-regulation of SIRT1 activity, while EX527 treatment reduced it. Cells were pretreated with 150 μM EX527/vehicle for 6 h and the cultures in fresh media were then exposed to 1500 lx light for 72 h. SIRT1 activity in the nuclear extracts was measured using the Epigenase Universal SIRT Activity Assay Kit, since SIRT1 mainly locates in nucleus. Scramble: cells were transfected with scrambled shRNA as negative controls; mTOR KD: cells with mTOR knockdown; PARP-1 KD: cells with PARP-1 knockdown; Lt: 1500 lx light exposure for 72 h; EX527: a SIRT1 inhibitor. All experiments were repeated in triplicate and the results are shown as the means ± SEM (**: *P* < 0.01, ***: *P* < 0.001)

## Data Availability

All data generated or analyzed during this study are included in this published article.

## Abbreviations

3AB: 3-Aminobenzamide
4EBP1: Eukaryotic initiation factor 4E-binding protein 1
AIF: Apoptosis-inducing factor
Akt: Protein kinase B
AMD: Age-related macular degeneration
ATP: 5′-Adenylate triphosphate
DMEM: Dulbecco Modified Eagle Medium
H & E staining: Hematoxylin-eosin staining
HEK293T: Human embryonic kidney 293 T
HUVECS: Human umbilical vein endothelial cells
KD: Knockdown
MEFs: Mouse embryonic fibroblasts
MOMP: Mitochondrial outer membrane permeabilization
mTOR: Mammalian/mechanistic Target of Rapamycin
mTORC1: Mammalian Target of Rapamycin Complex 1
mTORC2: Mammalian Target of Rapamycin Complex 2
NAD^+^: Nicotinamide adenine dinucleotide
NAMPT: Nicotinamide phosphoribosyltransferase
NSCLS: Non-small cell lung cancer
ONL: Outer nuclear layer
P23H RHO: Histidine at position 23 of the rhodopsin gene
PAR: Poly (ADP-ribose)
PARP-1: Poly (ADP-ribose) polymerase-1
PFA: Paraformaldehyde
PI: Propidium iodide
PMSF: Phenylmethanesulfonyl
POS: Photoreceptor outer segment
PTEN: Phosphatase and Tensin Homolog
rd1: Retinal degeneration 1
RGC: Retinal ganglion cells
ROS: Reactive oxygen species
RP: Retinitis pigmentosa
RPE: Retinal pigment epithelial cells
RSV: Resveratrol
S6K1: p70 ribosomal S6 kinase
shRNA: Short hairpin RNA
SIRT1: Sirtuin 1
TBS-T: Tris-buffered saline with 0.1% Tween-20
TSC2: Tuberous sclerosis complex 2

## References

[1] Zhu Y-X, Yao J, Liu C, Hu H-T, Li X-M, Ge H-M (2018). Long non-coding RNA MEG3 silencing protects against light-induced retinal degeneration. Biochem Biophys Res Commun.

[2] Paskowitz DM, LaVail MM, Duncan JL (2006). Light and inherited retinal degeneration. Br J Ophthalmol.

[3] Rose K, Walston ST, Chen J (2017). Separation of photoreceptor cell compartments in mouse retina for protein analysis. Mol Neurodegeneration.

[4] Contín MA, Benedetto MM, Quinteros-Quintana ML, Guido ME (2016). Light pollution: the possible consequences of excessive illumination on retina. Eye (London, England).

[5] Palczewski K, Kumasaka T, Hori T, Behnke CA, Motoshima H, Fox BA (2000). Crystal structure of rhodopsin: a G protein-coupled receptor. Science.

[6] Xue L, Zeng Y, Li Q, Li Y, Li Z, Xu H (2017). Transplanted olfactory ensheathing cells restore retinal function in a rat model of light-induced retinal damage by inhibiting oxidative stress. Oncotarget.

[7] Organisciak DT, Vaughan DK (2010). Retinal light damage: mechanisms and protection. Prog Retin Eye Res.

[8] Marc RE, Jones BW, Watt CB, Vazquez-Chona F, Vaughan DK, Organisciak DT (2008). Extreme retinal remodeling triggered by light damage: implications for age related macular degeneration. Mol Vis.

[9] Hirakawa M, Tanaka M, Tanaka Y, Okubo A, Koriyama C, Tsuji M (2008). Age-related maculopathy and sunlight exposure evaluated by objective measurement. Br J Ophthalmol.

[10] Farnoodian M, Wang S, Dietz J, Nickells RW, Sorenson CM, Sheibani N (2017). Negative regulators of angiogenesis: important targets for treatment of exudative AMD. Clin Sci (London, England : 1979).

[11] Lambert NG, ElShelmani H, Singh MK, Mansergh FC, Wride MA, Padilla M (2016). Risk factors and biomarkers of age-related macular degeneration. Prog Retin Eye Res.

[12] Taylor HR, Muñoz B, West S, Bressler NM, Bressler SB, Rosenthal FS (1990). Visible light and risk of age-related macular degeneration. Trans Am Ophthalmol Soc.

[13] Zhou H, Zhang H, Yu A, Xie J (2018). Association between sunlight exposure and risk of age-related macular degeneration: a meta-analysis. BMC Ophthalmol.

[14] Tsuruma K, Shimazaki H, Ohno Y, Inoue Y, Honda A, Imai S (2012). Metallothionein-III deficiency exacerbates light-induced retinal DegenerationMetallothionein-III deficiency. Invest Ophthalmol Vis Sci.

[15] Hunter JJ, Morgan JIW, Merigan WH, Sliney DH, Sparrow JR, Williams DR (2012). The susceptibility of the retina to photochemical damage from visible light. Prog Retin Eye Res.

[16] Kevany BM, Palczewski K (2010). Phagocytosis of retinal rod and cone photoreceptors. Physiology (Bethesda, Md).

[17] Thomas C, Ji Y, Wu C, Datz H, Boyle C, MacLeod B (2019). Hit and run versus long-term activation of PARP-1 by its different domains fine-tunes nuclear processes. Proc Natl Acad Sci U S A.

[18] Krietsch J, Caron M-C, Gagné J-P, Ethier C, Vignard J, Vincent M (2012). PARP activation regulates the RNA-binding protein NONO in the DNA damage response to DNA double-strand breaks. Nucleic Acids Res.

[19] Huang C-T, Huang D-Y, Hu C-J, Wu D, Lin W-W (2014). Energy adaptive response during parthanatos is enhanced by PD98059 and involves mitochondrial function but not autophagy induction. Biochim Biophys Acta (BBA) - Mol Cell Res.

[20] Paquet-Durand F, Silva J, Talukdar T, Johnson LE, Azadi S, van Veen T (2007). Excessive activation of poly (ADP-ribose) polymerase contributes to inherited photoreceptor degeneration in the retinal degeneration 1 mouse. J Neurosci.

[21] Kaur J, Mencl S, Sahaboglu A, Farinelli P, van Veen T, Zrenner E (2011). Calpain and PARP activation during photoreceptor cell death in P23H and S334ter rhodopsin mutant rats. PLoS One.

[22] Andrabi SA, Dawson TM, Dawson VL (2008). Mitochondrial and nuclear cross talk in cell death: parthanatos. Ann N Y Acad Sci.

[23] Wang Y, Dawson VL, Dawson TM (2009). Poly (ADP-ribose) signals to mitochondrial AIF: a key event in parthanatos. Exp Neurol.

[24] Fatokun AA, Dawson VL, Dawson TM (2014). Parthanatos: mitochondrial-linked mechanisms and therapeutic opportunities. Br J Pharmacol.

[25] Otera H, Ohsakaya S, Nagaura Z-I, Ishihara N, Mihara K (2005). Export of mitochondrial AIF in response to proapoptotic stimuli depends on processing at the intermembrane space. EMBO J.

[26] Polster BM, Basañez G, Etxebarria A, Hardwick JM, Nicholls DG (2005). Calpain I induces cleavage and release of apoptosis-inducing factor from isolated mitochondria. J Biol Chem.

[27] Floyd S, Favre C, Lasorsa FM, Leahy M, Trigiante G, Stroebel P (2007). The insulin-like growth factor-I-mTOR signaling pathway induces the mitochondrial pyrimidine nucleotide carrier to promote cell growth. Mol Biol Cell.

[28] Good DW, George T, Watts BA (2008). Nerve growth factor inhibits Na+/H+ exchange and formula absorption through parallel phosphatidylinositol 3-kinase-mTOR and ERK pathways in thick ascending limb. J Biol Chem.

[29] Benjamin D, Colombi M, Moroni C, Hall MN (2011). Rapamycin passes the torch: a new generation of mTOR inhibitors. Nat Rev Drug Discov.

[30] Sulaimanov N, Klose M, Busch H, Boerries M (2017). Understanding the mTOR signaling pathway via mathematical modeling. Wiley Interdiscip Rev.

[31] Chong ZZ, Shang YC, Wang S, Maiese K (2012). Shedding new light on neurodegenerative diseases through the mammalian target of rapamycin. Prog Neurobiol.

[32] Sinha D, Valapala M, Shang P, Hose S, Grebe R, Lutty GA (2016). Lysosomes: regulators of autophagy in the retinal pigmented epithelium. Exp Eye Res.

[33] Nakahara T, Morita A, Yagasaki R, Mori A, Sakamoto K (2017). Mammalian target of Rapamycin (mTOR) as a potential therapeutic target in pathological ocular angiogenesis. Biol Pharm Bull.

[34] Zhao D, Yang J, Yang L (2017). Insights for Oxidative Stress and mTOR Signaling in Myocardial Ischemia/Reperfusion Injury under Diabetes. Oxid Med Cell Longev.

[35] Fan B, Li F-Q, Zuo L, Li G-Y (2016). mTOR inhibition attenuates glucose deprivation-induced death in photoreceptors via suppressing a mitochondria-dependent apoptotic pathway. Neurochem Int.

[36] Besirli CG, Zheng Q-D, Reed DM, Zacks DN (2012). ERK-mediated activation of Fas apoptotic inhibitory molecule 2 (Faim2) prevents apoptosis of 661W cells in a model of detachment-induced photoreceptor cell death. PLoS One.

[37] Nakanishi T, Shimazawa M, Sugitani S, Kudo T, Imai S, Inokuchi Y (2013). Role of endoplasmic reticulum stress in light-induced photoreceptor degeneration in mice. J Neurochem.

[38] Chen W-J, Wu C, Xu Z, Kuse Y, Hara H, Duh EJ (2017). Nrf2 protects photoreceptor cells from photo-oxidative stress induced by blue light. Exp Eye Res.

[39] Liu S-Y, Song J-Y, Fan B, Wang Y, Pan Y-R, Che L (2018). Resveratrol protects photoreceptors by blocking caspase- and PARP-dependent cell death pathways. Free Radic Biol Med.

[40] Wood JPM, Lascaratos G, Bron AJ, Osborne NN (2007). The influence of visible light exposure on cultured RGC-5 cells. Mol Vis.

[41] Wang J, Du X-X, Jiang H, Xie J-X (2009). Curcumin attenuates 6-hydroxydopamine-induced cytotoxicity by anti-oxidation and nuclear factor-kappaB modulation in MES23.5 cells. Biochem Pharmacol.

[42] Takahashi K, Shimazawa M, Izawa H, Inoue Y, Kuse Y, Hara H (2017). Platelet-derived growth factor-BB lessens light-induced rod photoreceptor damage in MicePDGF-BB on light-induced retinal damage. Invest Ophthalmol Vis Sci.

[43] Shibagaki K, Okamoto K, Katsuta O, Nakamura M (2015). Beneficial protective effect of pramipexole on light-induced retinal damage in mice. Exp Eye Res.

[44] Martín-Oliva D, Martín-Guerrero SM, Matia-González AM, Ferrer-Martín RM, Martín-Estebané M, Carrasco M-C (2015). DNA damage, poly (ADP-ribose) polymerase activation, and phosphorylated histone H2AX expression during postnatal retina development in C57BL/6 MousePostnatal retinal cell DNA damage and death. Invest Ophthalmol Vis Sci.

[45] Strickfaden H, McDonald D, Kruhlak MJ, Haince J-F, Th'ng JPH, Rouleau M (2016). Poly (ADP-ribosyl)ation-dependent transient chromatin Decondensation and histone displacement following laser microirradiation. J Biol Chem.

[46] Hou W-H, Chen S-H, Yu X (2019). Poly-ADP ribosylation in DNA damage response and cancer therapy. Mutation Research/Reviews in Mutation Research.

[47] Donovan M, Carmody RJ, Cotter TG (2001). Light-induced Photoreceptor Apoptosis in vivo is Caspase Independent and Mediated by Nitric Oxide. Sci World J.

[48] Recchia AG, Musti AM, Lanzino M, Panno ML, Turano E, Zumpano R (2009). A cross-talk between the androgen receptor and the epidermal growth factor receptor leads to p38MAPK-dependent activation of mTOR and cyclinD1 expression in prostate and lung cancer cells. Int J Biochem Cell Biol.

[49] Maiese K (2017). Moving to the rhythm with clock (circadian) genes, autophagy, mTOR, and SIRT1 in degenerative disease and Cancer. Curr Neurovasc Res.

[50] Malagelada C, Jin ZH, Jackson-Lewis V, Przedborski S, Greene LA (2010). Rapamycin protects against neuron death in in vitro and in vivo models of Parkinson's disease. J Neurosci.

[51] Lafay-Chebassier C, Pérault-Pochat MC, Page G, Bilan AR, Damjanac M, Pain S (2006). The immunosuppressant rapamycin exacerbates neurotoxicity of Aβ peptide. J Neurosci Res.

[52] Shang YC, Chong ZZ, Wang S, Maiese K (2012). Prevention of β-amyloid degeneration of microglia by erythropoietin depends on Wnt1, the PI 3-K/mTOR pathway, bad, and Bcl-xL. Aging.

[53] Kurimoto T, Yin Y, Omura K, Gilbert H-Y, Kim D, Cen L-P (2010). Long-distance axon regeneration in the mature optic nerve: contributions of oncomodulin, cAMP, and pten gene deletion. J Neurosci.

[54] Houtkooper RH, Pirinen E, Auwerx J (2012). Sirtuins as regulators of metabolism and healthspan. Nat Rev Mol Cell Biol.

[55] Nakagawa T, Guarente L (2011). Sirtuins at a glance. J Cell Sci.

[56] Beneke S, Diefenbach J, Bürkle A (2004). Poly (ADP-ribosyl) ation inhibitors: promising drug candidates for a wide variety of pathophysiologic conditions. Int J Cancer.

[57] Houtkooper RH, Cantó C, Wanders RJ, Auwerx J (2010). The secret life of NAD+: an old metabolite controlling new metabolic signaling pathways. Endocr Rev.

[58] Rajamohan SB, Pillai VB, Gupta M, Sundaresan NR, Birukov KG, Samant S (2009). SIRT1 promotes cell survival under stress by deacetylation-dependent deactivation of poly (ADP-ribose) polymerase 1. Mol Cell Biol.

[59] Pillai JB, Gupta M, Rajamohan SB, Lang R, Raman J, Gupta MP (2006). Poly (ADP-ribose) polymerase-1-deficient mice are protected from angiotensin II-induced cardiac hypertrophy. Am J Phys Heart Circ Phys.

[60] Zhang J (2003). Are poly (ADP-ribosyl) ation by PARP-1 and deacetylation by Sir2 linked?. BioEssays.

[61] Jadeja RN, Powell FL, Jones MA, Fuller J, Joseph E, Thounaojam MC (2018). Loss of NAMPT in aging retinal pigment epithelium reduces NAD(+) availability and promotes cellular senescence. Aging.

[62] Bai P, Cantó C, Oudart H, Brunyánszki A, Cen Y, Thomas C (2011). PARP-1 inhibition increases mitochondrial metabolism through SIRT1 activation. Cell Metab.

[63] Zhang S, Cai G, Fu B, Feng Z, Ding R, Bai X (2012). SIRT1 is required for the effects of rapamycin on high glucose-inducing mesangial cells senescence. Mech Ageing Dev.

[64] Back JH, Rezvani HR, Zhu Y, Guyonnet-Duperat V, Athar M, Ratner D (2011). Cancer cell survival following DNA damage-mediated premature senescence is regulated by mammalian target of rapamycin (mTOR)-dependent inhibition of sirtuin 1. J Biol Chem.

[65] Cantó C, Sauve AA, Bai P (2013). Crosstalk between poly (ADP-ribose) polymerase and sirtuin enzymes. Mol Asp Med.

[66] Ghosh HS, McBurney M, Robbins PD (2010). SIRT1 negatively regulates the mammalian target of rapamycin. PLoS One.

