# Supplementary figures and images for: mTOR may interact with PARP-1 to regulate visible light-induced parthanatos in photoreceptors
Source: Cell Commun Signal. 2020 Feb 17;18:27. doi: 10.1186/s12964-019-0498-0 (PMC7025415; doi:10.1186/s12964-019-0498-0)

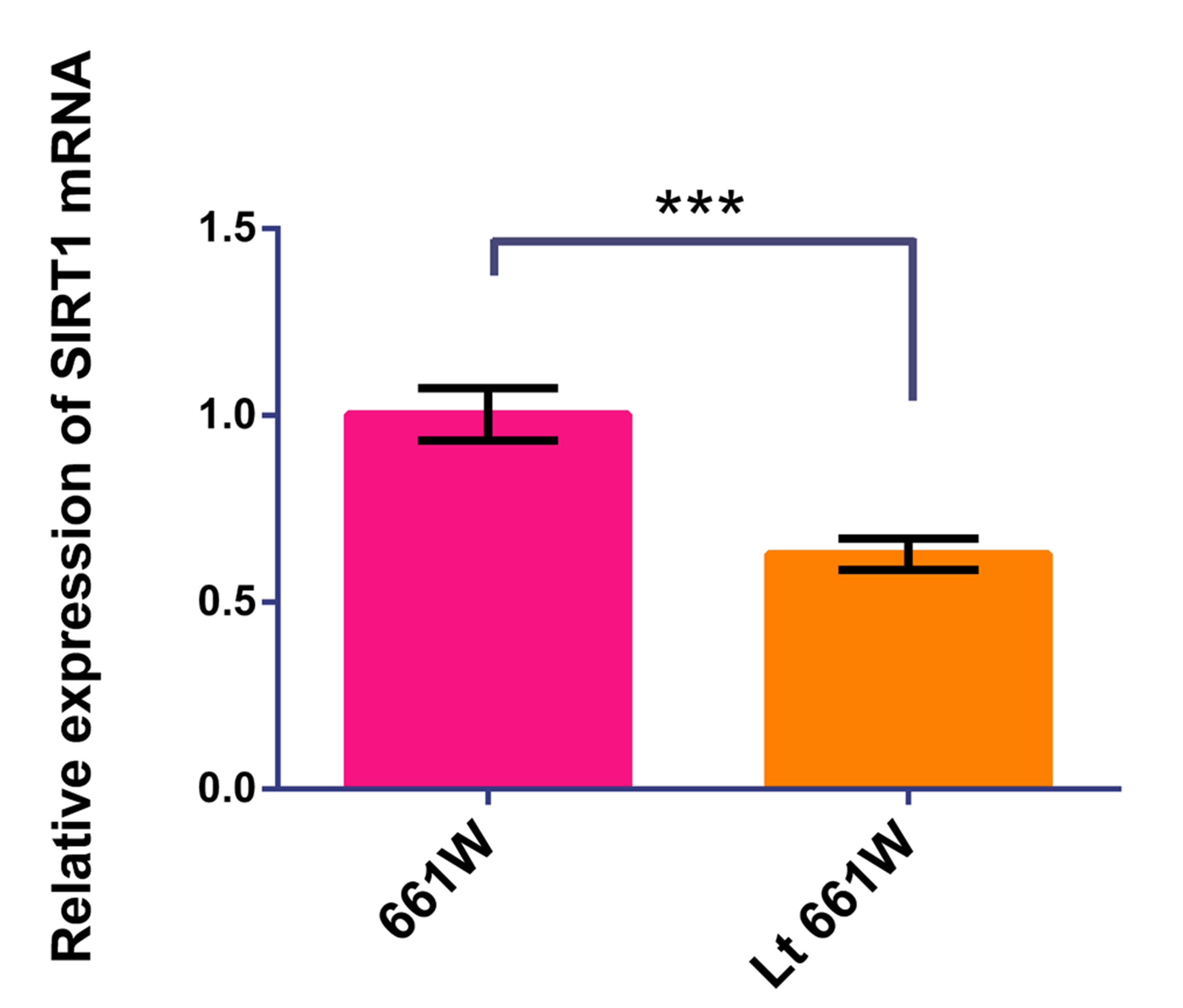

Supplement: Supplementary file 2 — Additional file 1. Supplementary Materials and Methods [file 12964_2019_498_MOESM2_ESM.zip › Supplementary Figure S1.tif]

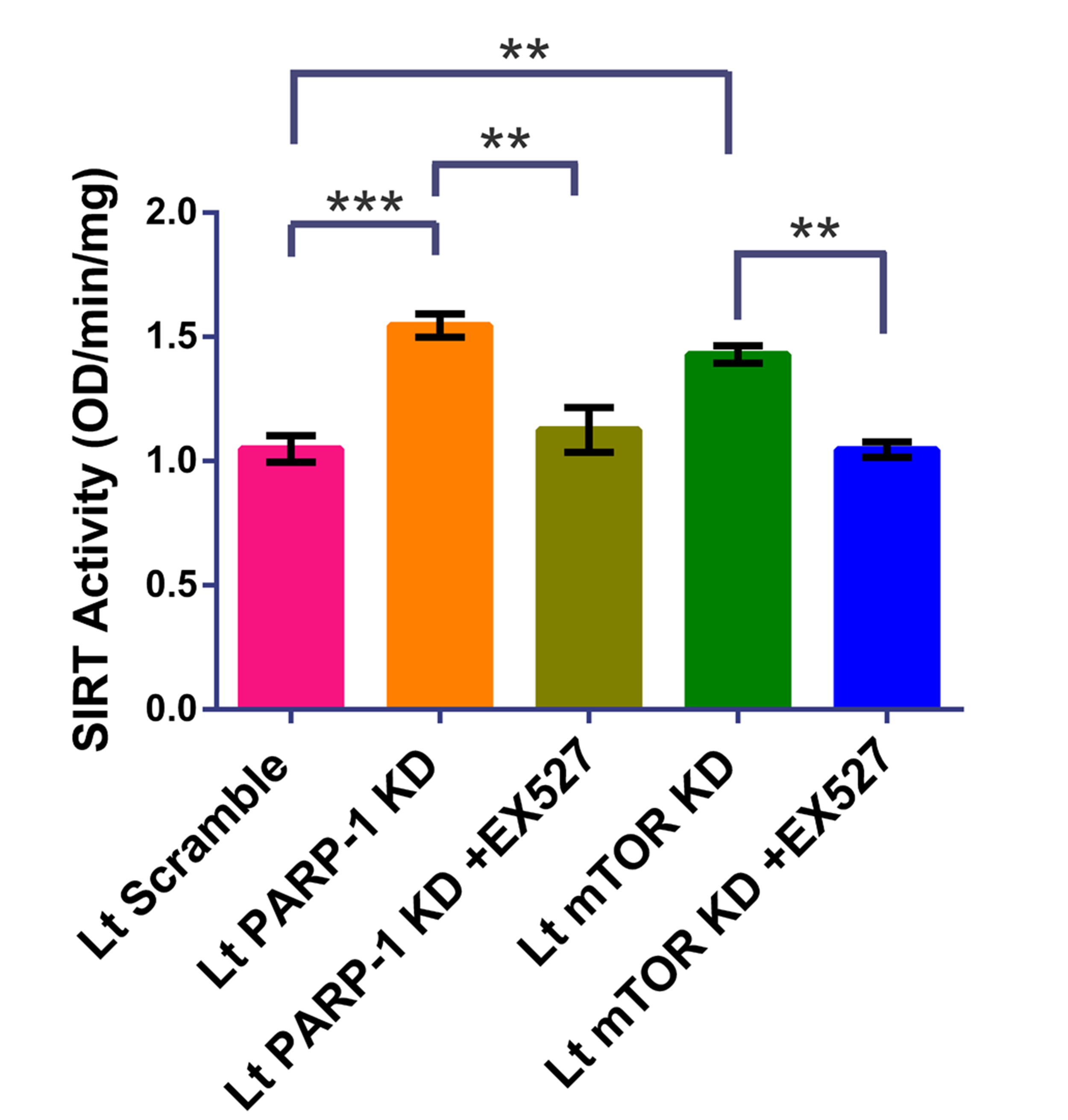

Supplement: Supplementary file 2 — Additional file 1. Supplementary Materials and Methods [file 12964_2019_498_MOESM2_ESM.zip › Supplementary Figure S2.tif]
