# Supplementary material for: mTOR may interact with PARP-1 to regulate visible light-induced parthanatos in photoreceptors
Source: Cell Commun Signal. 2020 Feb 17;18:27. doi: 10.1186/s12964-019-0498-0 (PMC7025415; doi:10.1186/s12964-019-0498-0)
Supplement: Supplementary file 3 — Additional file 2: Supplementary Figure Legends. Figure S1. SIRT1 mRNA levels decreased in light-damaged 661 W cells compared to control cells. Quantitative real-time PCR analysis of SIRT1 mRNA expression. β-ACTIN was used as an endogenous control. SIRT1 expression levels were normalized to the mean expression levels of β-ACTIN. Lt: 1500 lx light exposure for 72 h. All experiments were repeated in triplicate and the results are shown as the means ± SEM (***: P < 0.001). Figure S2. PARP-1 / mTOR knockdown caused up-regulation of SIRT1 activity, while EX527 treatment reduced it. Cells were pretreated with 150 μM EX527/vehicle for 6 h and the cultures in fresh media were then exposed to 1500 lx light for 72 h. SIRT1 activity in the nuclear extracts was measured using the Epigenase Universal SIRT Activity Assay Kit, since SIRT1 mainly locates in nucleus. Scramble: cells were transfected with scrambled shRNA as negative controls; mTOR KD: cells with mTOR knockdown; PARP-1 KD: cells with PARP-1 knockdown; Lt: 1500 lx light exposure for 72 h; EX527: a SIRT1 inhibitor. All experiments were repeated in triplicate and the results are shown as the means ± SEM (**: P < 0.01, ***: P < 0.001) [file 12964_2019_498_MOESM3_ESM.docx]

**Additional File 1: Supplementary Materials and Methods**

**Materials and methods**

**Quantitative real-time PCR assays**

Cells were seeded in six-well plates and cultured for 24 h. Sufficient fresh media were replaced before the light exposure treatment. After light exposure at 1,500 lx for 72 h, total RNA was extracted with RiboEx (GeneAll Biotechnology, Seoul, Korea Cat. No. 301-001) according to the manufacturer's protocol. RNA from each sample was reverse transcribed into single-stranded complementary DNA using a First Stand cDNA Synthesis Kit (Tiangen, Beijing, China Cat. No. KR211-02). Amplification and quantification were carried out in 20 μl reaction mixtures containing 10 μl SYBR Green Master Mix (TheroFisher, Shanghai, China Cat. No. 4309155), 1 μl cDNA, 4 μl of each primer, and 5 μl of ddH_2_O. The reaction conditions were as follows: 50°C for 2 min, 95°C for 2 min and then 40 cycles of 95°C for 15 s, 60°C for 15 s, and 72°C for 1 min. All reactions were performed in triplicate and β-ACTIN was used as the endogenous control. SIRT1 expression levels were normalized to the mean expression levels of β-ACTIN. Primers were purchased from Gene Copeia and corresponded to the following oligonucleotides:

SIRT1 (Cat. No. MQP032125)

Forward: TCTGAAAGTGAGACCAGTAGCA

Reverse: TGTAGATGAGGCAAAGGTTCCC

β-ACTIN (Cat. No. MQP026493)

Forward: CTAAGGCCAACCGTGAAAAG

Reverse: ACCAGAGGCATACAGGGACA

**SIRT1 activity assays**

The Epigenase Universal SIRT Activity Assay Kit (Epigentek, Brooklyn, NY, USA) was used to measure the deacetylase activity of SIRTs. Briefly, cells were seeded in six-well plates for 24 h. The cells were then pretreated with 150 μM EX527 / vehicle for 6 h prior to light exposure. Media were then replaced with fresh DMEM media. After light exposure at 1,500 lx for 72 h, nuclear extracts were isolated using the Nuclear and Cytoplasmic Protein Extraction Kit (Beyotime Biotechnology, Shanghai, China). A 5 μg aliquot of nuclear extract was then applied to microplate wells coated with an acetylated histone SIRT substrate. After incubation for 90 min, the SIRT-deacetylated products were recognized with a specific antibody. Finally, after adding the detection antibody and color developing solution, the absorbance at 450 nm was measured with a microplate reader to quantify SIRT1 activity.
